# Adaptive Optics Image Analysis Using Generative AI (GPT-4) Scripting: Exploratory Study

**DOI:** 10.2196/94906

**Published:** 2026-09-14

**Authors:** Jeroen AAH Pas, Ludo C van der Zanden, Yoeri van Leeuwen, Yara TE Lechanteur, Carel B Hoyng, Thomas Theelen

**Affiliations:** 1 Department of Ophthalmology Radboud University Medical Center Nijmegen, Gelderland The Netherlands; 2 Donders Institute for Brain, Cognition and Behaviour Nijmegen, Gelderland The Netherlands

**Keywords:** adaptive optics, generative artificial intelligence, generative AI, Stargardt disease, retina, imaging

## Abstract

**Background:**

The availability of dedicated image analysis scripts for adaptive optics (AO)–flood illumination ophthalmoscopy (FIO) is limited, especially for large-scale measurements and analyses of nonhealthy images. This limitation highlights the need for alternative approaches to facilitate automated and scalable analysis. Large language models may help develop such scripts.

**Objective:**

This study aimed to generate an analysis script for AO-FIO images in the R programming language using a widely available generative AI (GenAI; specifically, GPT-4) as a proof of principle for generating a functional but nonvalidated script.

**Methods:**

GPT-4 was used to generate an R script for the analysis of AO-FIO images. The code generated by GPT-4 was fine-tuned iteratively based on trial and error, testing the script for image preprocessing and analysis using images from 4 participants, including 1 healthy individual and 3 patients with Stargardt disease. The script code was subsequently checked for errors by another researcher who was naive to previous coding, using a different test set of AO-FIO images from 4 other participants (n=1 healthy individual and n=3 patients with Stargardt disease). The cone counts from 5 AO image snippets were compared with the counts independently recorded by 2 human graders and those generated by pre-existing AO analysis software that was trained on healthy participants.

**Results:**

After 54 iterations of instructions, a functional R script for the analysis of AO-FIO images was generated. The script identified and quantified blobs.

**Conclusions:**

We developed a preliminary nonvalidated script for cone detection in retinal AO images with support from GPT-4. Before such a script can be used in clinical research, it should undergo more fine-tuning and extensive testing. Future work should enhance the image analysis capabilities of the script and validate its results to assess the potential of AO-based cone counts as biomarkers in clinical trials.

## Introduction

The rapid evolution of modern imaging techniques has greatly advanced retinal scientific research. Converting these images into numeric data that can be used for analysis often requires scripts. Custom-made scripts for advanced image analysis software, regardless of the image modality, are not widely available, potentially leading to suboptimal interpretation and analysis of these images. Generative AI (GenAI), such as GPT-4, may be used for code generation and to generate and use code for image analysis [1].

One important advancement in retinal imaging is adaptive optics (AO)–flood illumination ophthalmoscopy (FIO), which allows high-resolution imaging of cone photoreceptors in the retina [2-4]. Cone counts measured on AO-FIO images are a promising new end point for clinical trials in inherited retinal diseases, such as Stargardt disease [5]. In Stargardt disease, cone photoreceptor degeneration occurs due to the accumulation of lipofuscin in the outer segments and the retinal pigment epithelium (RPE) [6,7]. Recent studies using AO imaging have shown that the cone photoreceptor structure is altered in Stargardt disease. In the unaffected retina of patients with Stargardt disease, the cone mosaic is unchanged. However, in the affected retina, a disturbed photoreceptor mosaic, dark non-waveguiding cones, enlarged cones near the fovea, and discrete hyper-reflective foci have been described, as well as a decrease in photoreceptor count [8-11]. This decline in cone photoreceptor count may serve as a biomarker in the future, thereby enabling researchers to study the effect of potential therapies at the cone photoreceptor level [12-14].

Considering cone count as a possible biomarker in clinical trials, AO analysis software is urgently needed [15]. Unfortunately, to date, there are no data on the reliability and repeatability of the available AO software provided by the manufacturer of the only Conformité Européenne (CE)–marked AO camera (Aodetect; Imagine Eyes), especially for eyes with significant retinal pathology. In addition, this application can only be applied manually for cone analysis within small areas of 0.21°×0.21° in a 4°×4° image, which makes it less useful for large-scale data analysis.

In addition to the AO analysis software provided by the manufacturer, other software for automated cone detection has been developed. Valterova et al [16] designed a tool for automated cone density calculation based on montaged AO images. Wooning et al [17] developed a deep learning–based model for detecting cone photoreceptor cells in single AO images, outperforming the manufacturer’s autodetection software. However, these models were trained only on healthy participants, and their use on pathological images has not yet been evaluated.

For AO scanning light ophthalmoscope (SLO), a different type of AO imaging, automated models for cone detection have been developed for both confocal and split-detector images and are trained and reliable for detecting cones not only in the retina of healthy participants [18,19] but also in those of both healthy participants and patients with Stargardt disease [20]. Although yielding promising results, photoreceptors may have different optical properties on AO-SLO images compared to AO-FIO images [3,21], potentially reducing the reliability of such scripts.

In this study, we explored the feasibility of performing AO image analysis using a script generated with GPT-4. With this approach, we aim to take the initial steps toward developing a new open-access cone count analysis script for AO images.

## Methods

### Study Design and Participants

This study was designed as a proof-of-principle study. AO images of 2 healthy participants (3 eyes; with no history of ocular diseases and no retinal abnormalities) and 6 patients (12 eyes) with genetically confirmed Stargardt disease were included in this study. The patients with Stargardt disease all had significant macular pathology, but no disease affecting the peripapillary region or spreading outside of the vascular arch. Two test sets of AO-FIO images made with the RTX1 AO retinal camera were created. Each test set included AO-FIO images from a total of 4 participants, including 1 healthy individual and 3 patients with Stargardt disease. The training set included 118 images (32 healthy); the test set contained 157 images (38 healthy).

### Data Collection

The images were acquired during outpatient clinic visits. Images were taken starting at the optic nerve head, then moving temporally toward the foveal region and the temporal retina. Image acquisition settings were optimized for each individual image. No formal image quality criteria were used.

### Ethical Considerations

All images used in this study were derived from the Radboudumc ophthalmology biobank, which was approved by the Radboudumc Ethics Committee (Commissie Mensgebonden Onderzoek; CMO; 2017-3535). The study was conducted in adherence with the provisions of the Declaration of Helsinki, and all study participants gave written informed consent prior to inclusion in the study.

### Automated Blob Detection

On AO-FIO images, photoreceptors generally appear as a mosaic of bright maxima [4], resulting in grayscale retinal images (4°×4°) consisting of bright blobs, that is, round groups of pixels with similar pixel intensities standing out against the generalized background gray level. Thus, retinal photoreceptors on AO images can be counted using blob detection analysis [22], which eventually allows estimation of the cone density within the examined area of the retina. Manual counting of the blobs is not practical, as there may be >10,000 blobs per image, resulting in a highly time-consuming analysis if performed by human graders. Automated analysis techniques, on the other hand, are expected to perform with much higher speed and possibly higher accuracy.

### Development of the Automated Blob Detection Script

One of the authors (JAAHP) used GPT-4 (OpenAI; used between January 22, 2024, and February 19, 2024) to generate a script in the R programming language (version 4.3.2; R Foundation for Statistical Computing) for automated blob counting. ChatGPT was used through OpenAI’s hosted service. The underlying computational environment is managed by OpenAI and is not exposed to the user; therefore, detailed hardware and software specifications are not available. RStudio (version 2022.02.1; Posit Software PBC) was used to run the script. The following R packages were used in the script: *EBImage* (version 4.44.0) [23], *imager* (version 0.45.2) [24], *abind* (version 1.4-5) [25], and *spatstat* (version 3.0-7) [26]. Only textual instructions were given to the GenAI while generating the code. Using the input provided by GPT-4, preprocessing steps were built into the code for accurate analysis. After every preprocessing step, the code returned the processed image, allowing the researchers to verify whether all preprocessing steps were applied as intended to the image. The initial code was then tested using images from test set 1, and the programming errors that occurred were reported back to GPT-4. In this way, the model was facilitated to generate code while iteratively eliminating programming errors during the process.

To ensure the efficacy of the blob analysis, the language model was directed to generate code that highlighted the identified cones on the original images. Subsequently, the accurate location of the blobs on the altered image was manually verified to ensure that only genuine blob-like structures, and not vessels or background grayness, were indicated. This facilitated visual verification by the researchers (JAAHP, LCVDZ, and TT) that the code correctly identified blobs.

### Proof-of-Principle Evaluation of the Generated Script

Next, another author (LCVDZ) ran the code using a test set of AO-FIO images different from the training data to evaluate the performance of the script on independent images.

To test the script’s performance, the cone counts of 5 AO images (Figure 1) obtained from 2 healthy eyes and 3 eyes of patients with Stargardt disease, selected from the test set, were manually assessed by 2 independent graders (JAAHP and YVL) and compared with those generated by the script and the AO box tool of Wooning et al [17].

**Figure 1:**
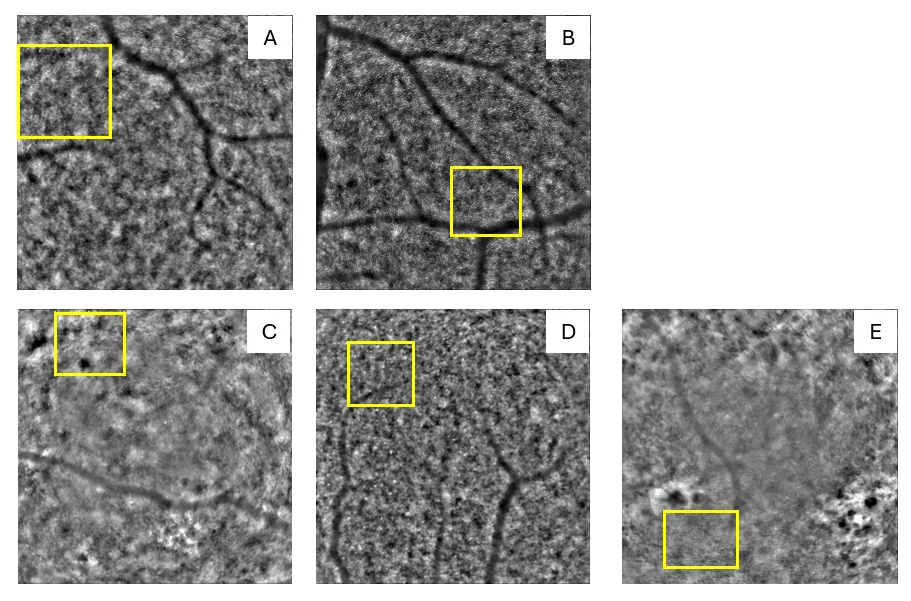
Original adaptive optics images from which the analyzed image snippets (yellow boxes) were extracted. Images A and B are from a healthy participant and show a clear mosaic of bright dots and blood vessels. Images C, D, and E are from patients with Stargardt disease and show an altered (D) or absent (C and E) bright-dot mosaic. Image A was acquired 2° from the fovea; image B, 6° from the fovea; image C, 6° from the fovea; image D, 6° from the fovea; and image E, 4° from the fovea.

Each AO image covered a 4°×4° field of view, which we considered too large for full manual annotation in a single step. Therefore, we extracted smaller regions of interest at random locations within each selected AO image. For the manual grading, the selected image snippets were uploaded to ImageJ (version 1.53t; National Institutes of Health). Using the multiselection tool, all round, bright blobs were counted by the 2 graders. Both graders had extensive experience (>1 year) with the AO RTX1 camera, including expertise in AO image interpretation. No formal grading training was conducted. Grading was performed independently, and the graders were blinded to the presence of pre-existing pathology. There was no adjudicated consensus label. Annotations were saved using the ROI Manager. The intraclass correlation coefficients between the graders, the script, and the AO box tool were calculated, and Bland-Altman analyses were performed. Additional validation of the script was not conducted, as the objective of this study was to show the proof of principle of the produced code.

## Results

Following a cumulative total of 54 commands given to GPT-4, a functional R script was achieved. At 3 distinct intervals, the code required manual modifications using snippets that had been previously generated by GPT-4: during the execution of the preprocessing steps, to complete the weighted cone counting, and to conclude the script. GPT-4 needed to be provided with an earlier version of the script on 3 occasions to maintain consistency. The final script was tested on all 118 images and did not produce any errors. The full script can be found in Multimedia Appendix 1, and the full prompting history is available through the link provided in Multimedia Appendix 2. The manual intervention in the automatic code creation is highlighted in bold in Multimedia Appendix 2. Table 1 shows the human modifications made during the script generation.

**Table 1:** Interventions and corresponding prompt numbers.

Prompt numbers	Human interventions
1	Feeding the idea of “blob detection analysis” to GPT-4
32, 39, and 43	Feeding GPT-4 with previously generated code to stay on track
35	Feeding GPT-4 with previously generated code. This code contained manually inserted preprocessing steps generated earlier in the prompting process (prompts 23 and 30). Additionally, code was added to save the processed image after each preprocessing step to check whether the step has been executed.
43 and 47	Steering GPT-4 in another direction because the previous steps (for counting overlapping blobs) did not work.
51	Manually extrapolating the code lines for counting overlapping blobs (blobs with an area larger than x pixels).
≥54	Finishing the code, implementing the data frame for reading files from a folder, and printing data output lines generated in prompts 35 and 36.

No particular prompting techniques were used, as the researcher using the GenAI lacked substantial experience with prompting for code development. GPT-4 developed a method for image analysis, including preprocessing and thresholding of the images. The settings of the threshold levels had to be chosen manually by the researcher, although GPT-4 provided recommendations for identifying optimal threshold settings (refer to prompt 22 in Multimedia Appendix 2). Figure 2 shows a visual representation of the image analysis workflow, including the preprocessing steps recommended by GPT-4. These steps included contrast stretching, using the “normalize” and “equalize” functions, Gaussian blur, and thresholding itself. The values for the cone count were compiled into an R data frame to facilitate efficient analysis for research purposes.

**Figure 2:**
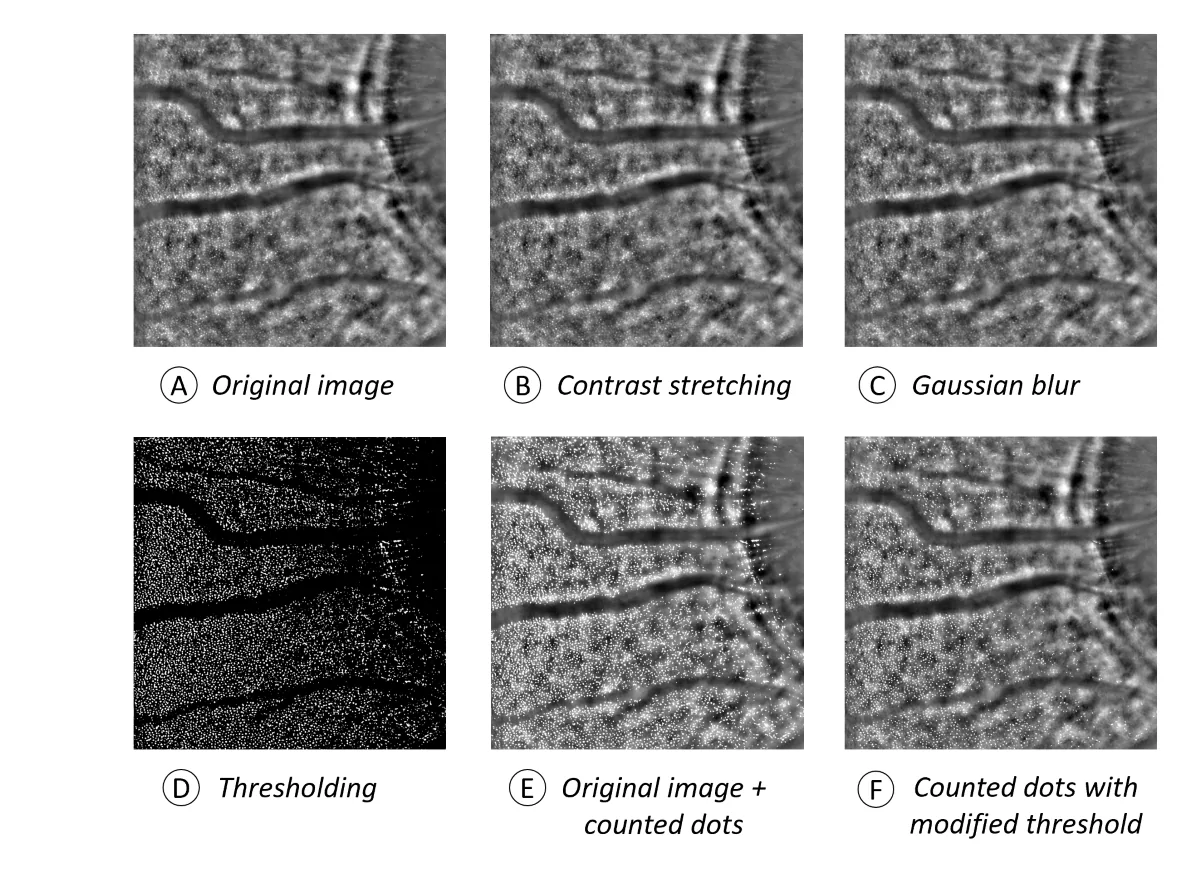
Image-processing workflow created by GPT-4. (A) Original image; (B) image after contrast enhancement using the “normalize” and “equalize” functions; (C) image after application of a Gaussian blur filter; (D) image generated after applying the default thresholding parameters; (E) detected blobs overlaid on the original image using default threshold settings; and (F) detected blobs overlaid on the original image after optimization of the detection parameters (width=4.5 pixels, height=4.5 pixels, and threshold=0.0043).

Following the initial testing phases, the threshold parameter (thresholding window w=4.5 and h=4.5; threshold=0.0043) was manually calibrated based on clearly overlooked and incorrectly identified blobs. A single threshold value was applied across all images. This threshold was determined based on the visual performance of the script on the training dataset. Figure 3 demonstrates the effect of different thresholds and the size of the local thresholding window (w and h).

**Figure 3:**
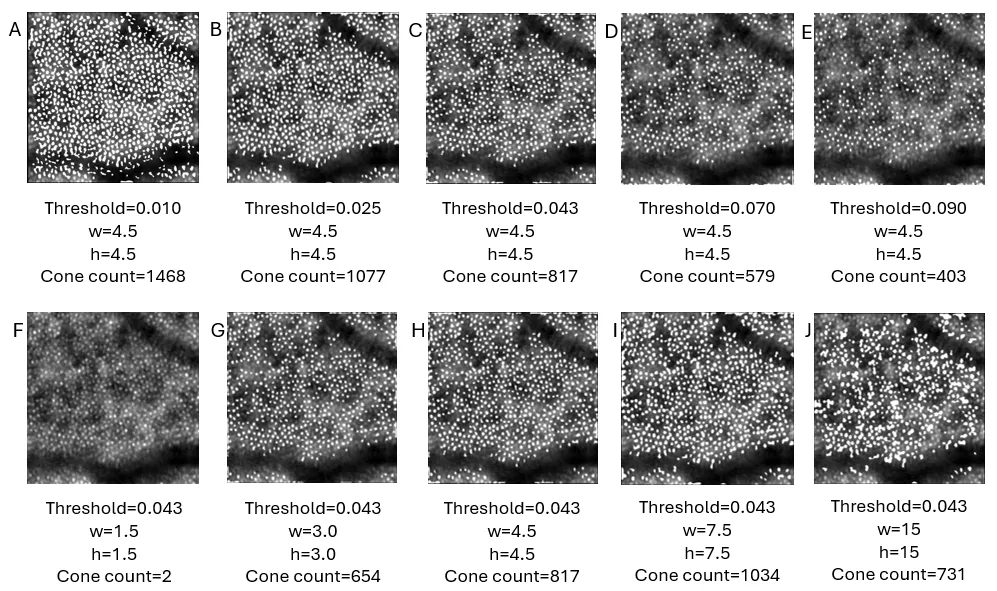
Effect of the threshold value and the thresholding window size on blob detection. For our analysis, we used a thresholding value of 0.043, a width (w) of 4.5 pixels, and a height (h) of 4.5 pixels. (A-E) When the thresholding value was increased while the thresholding window size was kept constant, fewer blobs were detected. (F-J) When the thresholding window size was increased while the thresholding value was kept constant, more blobs were detected until a maximum was reached, after which the blob count decreased again.

Subsequently, the algorithm underwent additional validation (conducted by LCVDZ) on test set 2, as outlined in the Methods section, to evaluate the stability and reliability of the developed code. The algorithm executed without issues, produced quantitative results for blob counting, and did not encounter any technical failures.

Next, the cone counts for the predefined AO image snippets (Figure 4) were calculated using the generated script and the AO box tool, and the images were graded by the 2 independent graders. The thresholding parameters determined during the testing phase, as mentioned previously, were used. The results of the cone measurements are shown in Table 2. The annotations generated by the script, the AO box, and the 2 graders are demonstrated in Multimedia Appendix 3.

**Figure 4:**
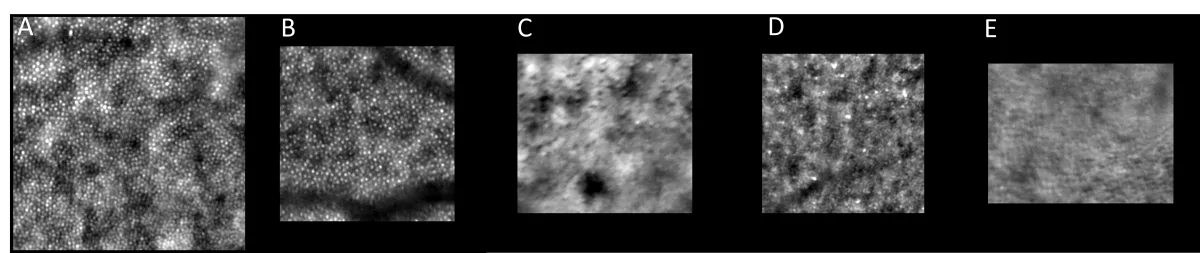
Image snippets used to compare the generated script with those of 2 independent graders. Snippets A and B were extracted from adaptive optics (AO) images of healthy participants and show a clear mosaic of bright, round dots. Snippets C, D, and E were extracted from AO images of patients with Stargardt disease. A clear mosaic of bright dots is not detectable in these images, suggesting altered photoreceptor reflectivity or photoreceptor defects.

**Table 2:** Cone counts per image calculated by the generated script and recorded by the 2 independent graders^a^.

Images	Script cone count	Grader 1 cone count	Grader 2 cone count	Adaptive optics box [17]	Difference between mean grader cone count and script cone count (%)
A	2062	1894	1963	2566	+6.9
B	817	747	728	1068	+10.8
C	60	48	35	1051	+44.6
D	571	223	215	272	+136.1
E	136	140	127	966	+1.8

^a^Agreement between grader 1 and grader 2 was nearly perfect. The intraclass correlation coefficient for absolute agreement was 0.999 (95% CI 0.992-1.000; *P*<.001).

A Bland-Altman analysis was conducted to further assess agreement between grader 1 and grader 2. The mean difference (bias) was −3.2, indicating that grader 1 recorded slightly lower cone values than grader 2 on average. The 95% limits of agreement ranged from −75.7 to 69. The plot is demonstrated in Figure 5.

**Figure 5:**
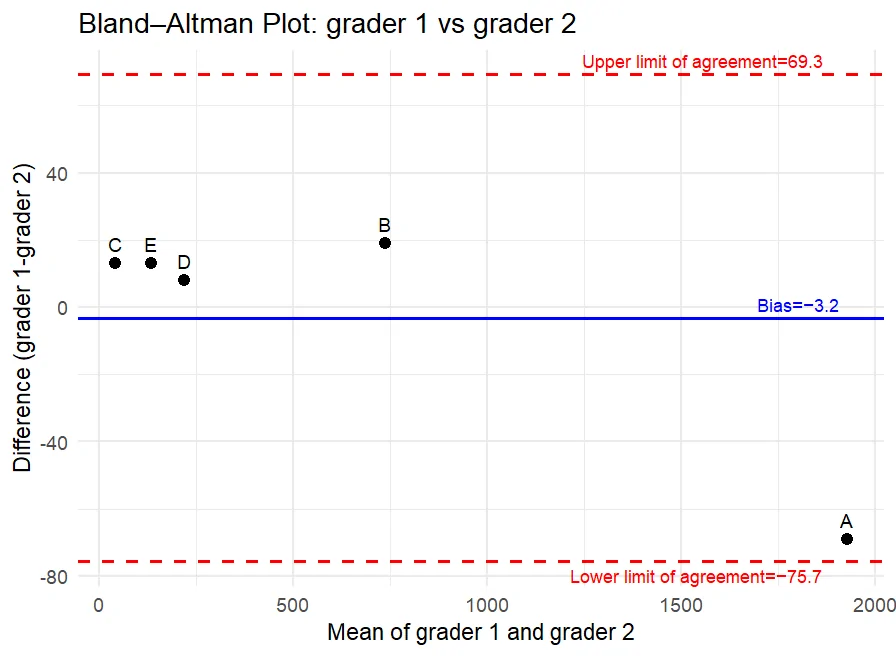
Bland-Altman plot comparing the cone count recorded by grader 1 and grader 2. The largest absolute difference in cone count between graders 1 and 2 was observed in image Figure 4A.

The mean of grader 1 and grader 2 was compared with the generated script (intraclass correlation coefficient=0.977, 95% CI 0.770-0.998; *P*<.001) and the AO box (intraclass correlation coefficient=0.724, 95% CI −0.105 to 0.967; *P*=.009). The script was also compared with the AO box (intraclass correlation coefficient=0.725, 95% CI −0.047 to 0.966; *P*=.03).

A Bland-Altman analysis comparing the mean grader values and the generated script scores revealed a mean bias of −117.2 (SD 138.4, indicating that the averaged human ratings generally produced lower cone counts than the generated script. The 95% limits of agreement ranged from −393.9 to 159.5.

A Bland-Altman analysis comparing the AO box and the generated script scores revealed a mean bias of 455.4 (SD 499.8), indicating that the AO box generally produced higher cone counts than the generated script. The 95% limits of agreement ranged from **−**544.2 to 1455.0. The Bland-Altman plots are shown in Multimedia Appendix 4.

## Discussion

### Principal Findings

In our current study, we used GenAI (GPT-4) to generate R-based image analysis code with the potential to perform quantitative analysis of retinal AO-FIO imaging. A key benefit of using GenAI in image analysis lies in its broad availability. This allows researchers to generate scripts for complex image analysis software without extensive pre-existing programming knowledge. However, it appears that using GenAI for image analysis still necessitates a basic understanding of image preprocessing, the principles of image analysis, and the use of GenAI (Multimedia Appendix 2). For instance, one should know the appropriate terminology for the appearance of a certain structure (here, photoreceptors as blobs) in image analysis. In both healthy patients and patients with Stargardt disease, the developed algorithm successfully identified blob-like structures most likely corresponding to photoreceptors throughout the imaging field. Comparing the script cone counts with those of the human graders demonstrated that the generated script counted more cones than the average count of the human graders in every image. In the healthy images A and B, which are depicted in Figure 4, it counted +6.9% (134 cones) and +10.8% (80 cones) more cones, respectively, than the human graders. The worst performance was observed on image D, where the script counted 136.1% more cones (352 cones) than the human graders, as it appears to have identified many false positives at the image border, as shown in Multimedia Appendix 3. The best apparent performance of the graded script was on image E, in which it counted +1.8% (4 cones) more than the average count of the graders.

The generated script counted fewer cones than the AO box tool in both images from healthy participants (506 and 257 fewer, respectively) and also counted fewer cones in Stargardt images C and E (991 and 830 fewer, respectively) in Multimedia Appendix 3, while counting more cones than the AO box in image D (299 more cones) in Multimedia Appendix 3. The intergrader agreement analysis demonstrated that the human graders had high agreement. The generated script also demonstrated high agreement with the mean of the human graders, but low agreement with the AO box. It should be noted that this analysis was performed on only 5 images; therefore, no conclusions can be drawn from these findings. However, it is noteworthy that the generated script had low agreement with the AO box. This may be because the AO box was trained only on healthy participants, whereas our script was generated and tuned using both healthy patients and patients with Stargardt disease.

Our script, for which the threshold values were manually determined using Stargardt images (excluding any of the tested images), was less likely to produce false positives when clear cones were visible. However, in images C and E in Multimedia Appendix 3, some of the selected cones appear to differ between the human graders and the generated script. Moreover, even the intergrader interpretation differed for these images. This is most likely due to the altered appearance of cone photoreceptors in Stargardt disease, which might be a major issue for cone counting. In this study, we used criteria similar to those applied in the AO box study by Wooning et al [17] for defining cone photoreceptors. We used the criterion that a cone photoreceptor should be a bright, round structure. They defined a photoreceptor according to the following criteria: the structure had to be round, exhibit an intensity peak, and have a similar size and spacing as neighboring structures. This last criterion was not used in our study. However, it is known that cone spacing changes in Stargardt disease [27], making this criterion for identifying a cone unreliable when counting cones in the pathological retina. Before AO can be used as an end point, or even as a secondary biomarker for clinical trials, there needs to be consensus on the definition of a cone in Stargardt disease to reliably count the number of cones.

### Limitations

This study has several limitations that warrant consideration when interpreting the results. The most significant limitation we encountered with R was the lack of options for watershed analysis. We circumvented this issue by estimating the cone count based on a predetermined cone pixel size definition. This option only works when all analyzed images have the same image resolution as the images in the training set. Nevertheless, watershed segmentation of the blobs would most likely have made the script more reliable [28]. Other quality measures that we wanted to implement in the script but that did not work were the width-to-height ratio for cones, as cones are often round, and Voronoi analysis to assess the cone mosaic [29]. In hindsight, R appeared not to have been the most logical choice of programming language for image analysis. We chose R because it was already available within our research environment and was more familiar to the team than other programming languages, such as Python. In retrospect, Python-based libraries may provide more mature and flexible tools for image analysis applications of this type.

Another weakness of the script we generated is the manually derived threshold value. For large-scale automated analysis of AO images, however, this value may need to be adapted to different datasets. Therefore, for the script to function reliably, an automated thresholding method will be required.

It should be noted that the script was generated using GPT-4. More advanced GenAI models are already available, potentially generating more sophisticated scripts. However, thorough testing and validation of such scripts will still be essential before using them in clinical research [30]. For now, we were able to generate a functional script for AO image analysis that was capable of detecting blob-like structures in retinal images. We did not develop or validate this script as a clinically robust research tool. Before such an approach could be considered suitable for clinical or large-scale research applications, the script would require substantial refinement, methodological validation, and review by experts in computer vision and biomedical image analysis to address issues related to generalizability, parameter optimization, and robustness across datasets. This study sought solely to demonstrate the feasibility of using GPT-4 for quantitative image analysis coding in AO imaging applications. The present study provides a preliminary evaluation of the generated script by comparing its output on 5 image snippets with counts obtained by human graders and the AO box tool. Although the script demonstrated strong agreement with the human graders, the small sample size and exploratory nature of this analysis preclude definitive conclusions regarding its accuracy, reliability, or generalizability. Therefore, a larger validation study using an improved version of the script and a substantially larger set of images is required before its performance can be comprehensively assessed and validated.

A general challenge in using GenAI is the occurrence of hallucinations [31]. Hallucinations, in the field of natural language processing, are defined as situations in which the generated product of GenAI is inconsistent with the given input or expected output environment, violates facts, or lacks meaning [32]. Hallucination in coding often appears when adding new coding snippets to perform a certain function in an already existing script or coding environment [33]. Adding medical images directly to ChatGPT, which we did not do, can also cause hallucinations, generating unreliable output [34]. While generating our AO image analysis script, we encountered some hallucination-related issues. As can be seen in Multimedia Appendix 2, GPT-4 claimed it could add watershed analysis and Voronoi analysis to the script, but this resulted only in a nonfunctioning script, which required us to reprompt using an older version of the script to stay on track. Extensive testing of the GenAI output is necessary to ensure that no hallucinated strings of code are present in a final script that will be used for formal analysis. Furthermore, researchers must be aware that GenAI may “forget” previous prompts, which may need reiteration, and that it may not be able to combine ideas from earlier failed steps with the current step. It has been shown that learning and applying prompt engineering [35] may lead to a reduced number of prompts needed. Another potential advantage of prompt engineering is that prompts should be designed in such a way that older generated code is not “forgotten” by GenAI. Standardized and improved prompting may result in more effective results with improved precision.

In conclusion, we were able to generate a functional script using GPT-4 that was able to perform blob detection in AO images and deliver plausible output compared to human graders and pre-existing software. However, before such a script can be used reliably in research, it should undergo extensive fine-tuning, testing, and validation to enhance its robustness. Future research should focus on more potent image analysis software coding and the validation of its results to ultimately assess the usability of cone counts measured on AO images as a biomarker for clinical trials.

## Data Availability

The final scripts and prompts are available in the multimedia appendices. Test images and outputs can be requested from the corresponding author.

## Appendix

Multimedia Appendix 1 R script generated with GPT-4.

Multimedia Appendix 2 Complete ChatGPT-prompting sequence (GPT-4).

Multimedia Appendix 3 Cone measurement annotations.

Multimedia Appendix 4 Bland-Altman plots comparing the mean grader cone count with the generated script cone count and the generated script cone count with the adaptive optics box cone count.

## Abbreviations

AO: adaptive optics
CE: Conformité Européenne
CMO: Commissie Mensgebonden Onderzoek
FIO: flood illumination ophthalmoscopy
GenAI: generative AI
RPE: retinal pigment epithelium
SLO: scanning light ophthalmoscope
