# Supplementary material for: Adaptive Optics Image Analysis Using Generative AI (GPT-4) Scripting: Exploratory Study
Source: JMIR Form Res. 2026 Sep 14;10:e94906. doi: 10.2196/94906 (PMC13620430; doi:10.2196/94906)
Supplement: Supplementary file 1 [file formative_v10i1e94906_app2.pdf]

Do you know blob detection

Prompt no.

1

Starting general to let the chatbot warm up

Yes, blob detection refers to a common image processing technique used to identify and extract information about "blobs" in an image. A blob is a region of an image that differs in properties, like brightness or color, compared to surrounding regions. Here's an overview:

Key Concepts:

1. Definition of Blobs:
   - Blobs are typically groups of connected pixels that share some common property, like intensity or color.
2. Applications:
   - Blob detection is widely used in various applications like object tracking, image segmentation, video analysis, and pattern recognition.
3. Techniques Used:
   - **Thresholding:** Separating objects from the background based on intensity.
   - **Edge Detection:** Identifying object boundaries.
   - **Filtering:** Using filters like Laplacian of Gaussian, Difference of Gaussians, etc., to enhance blob regions.
   - **Watershed Algorithm:** For segmenting touching objects.
4. Attributes of Blobs:
   - Size, shape, location, intensity, color, and texture.
5. Challenges:
   - Variability in size and shape of blobs, overlapping blobs, and varying lighting conditions can affect detection accuracy.
6. Software and Libraries:
   - Tools like OpenCV, MATLAB, and scikit-image in Python offer functions and algorithms for blob detection.

Blob detection algorithms typically involve scanning the image for regions that meet certain criteria (like brightness or color thresholds) and then grouping adjacent pixels together as a single blob. Advanced methods may include filtering techniques to remove noise and enhance the features of the blobs.

Is blob detection possible in R? 2

Yes, blob detection is indeed possible in R. While R is primarily known for its statistical and data analysis capabilities, it also has packages that can handle image processing tasks, including blob detection. Here are some steps and tools you can use for blob detection in R:

Tools and Packages:

1. EBImage:
   - EBImage is a Bioconductor package that provides general-purpose image processing and analysis capabilities. It can be used for tasks like image segmentation, which is a key part of blob detection.
2. imager:
   - Imager is another R package for image processing. It extends the magrittr syntax to make image processing operations more intuitive and R-like.

Steps for Blob Detection:

1. Install and Load Necessary Packages:
   - You would typically start by installing and loading the required packages, such as `EBImage` or `imager`.
2. Read the Image:
   - Load the image you want to process.
3. Preprocess the Image:
   - Depending on the nature of your image and the blobs, you might need to preprocess the image. This could include converting it to grayscale, normalizing, or thresholding.
4. Blob Detection:
   - Apply blob detection algorithms. This might involve finding regions of interest (ROI) in the image, which can be achieved through segmentation techniques.
   - In `EBImage`, functions like `thresh` (for thresholding), `bwlabel` (for labeling connected components), and `computeFeatures` (for extracting properties of blobs) can be useful.
5. Analyze and Extract Blob Information:
   - After detecting blobs, you can analyze them based on various properties like size, shape, and location.

Example in R:

R Code kopiëren

```
library(EBImage)

# Read the image
image <- readImage('path/to/image.jpg')

# Convert to grayscale and threshold
image <- channel(image, "gray")
threshImage <- thresh(image, w=10, h=10, offset=0.05)

# Detect blobs
blobs <- bwlabel(threshImage)

# Analyze blobs
features <- computeFeatures.shape(blobs)
print(features)
```

This is a simplified example. The actual process might require more fine-tuning depending on the specifics of your images and the nature of the blobs you're trying to detect.

What does the thresh function and the bw label functilon do? 3

In image processing within the R programming environment, particularly with the `EBImage` package, the `thresh` and `bwlabel` functions play specific roles:

1. `thresh` Function:
   - The `thresh` function is used for image thresholding. Thresholding is a technique to create a binary image from a grayscale image. It sets all the pixels in the image to either black or white, based on a threshold value.
   - In `EBImage`, `thresh` typically involves specifying a threshold value, which is used to decide whether a pixel should be turned black (usually representing the background) or white (representing the foreground or objects of interest).
   - The function can use different methods for calculating the threshold, which can be adapted based on the characteristics of the image.
2. `bwlabel` Function:
   - The `bwlabel` (Binary White Label) function is used for labeling connected components in a binary image. In other words, it identifies disjoint and connected regions (blobs) in a binary image and assigns a unique label to each region.
   - This function is crucial for blob analysis since it allows you to distinguish between different objects in an image and analyze each one separately.

Both of these functions are integral to many image processing tasks, especially in tasks like object detection and segmentation, where you first need to isolate objects of interest (using thresholding) and then identify and analyze each object separately (using connected component labeling).

I have an image with blobs, in gray scale, the blobs are white, about 25 pixels in surface area, and surroundend by a darker background. Can you provide me with a r code? 4

Certainly! To process an image with the characteristics you described (grayscale, white blobs about 25 pixels in area, on a darker background), we can use the `EBImage` package in R. The code will follow these steps:

1. Read the image.
2. Threshold the image to separate the blobs from the background.
3. Label the connected components (the blobs).
4. Filter out blobs based on size if necessary.

Here's a sample R code snippet for this process:

R Code kopiëren

```r
# Load the EBImage package
if (!requireNamespace("BiocManager", quietly = TRUE))
    install.packages("BiocManager")
BiocManager::install("EBImage")
library(EBImage)

# Step 1: Read the image
image <- readImage('path/to/your/image.jpg') # Replace with your image path

# Assuming the image is already in grayscale
# If not, convert to grayscale
image <- channel(image, "gray")

# Step 2: Threshold the image
# Adjust threshold value as needed. This is just an example.
threshImage <- thresh(image, w=10, h=10, offset=0.05)

# Step 3: Label the connected components
blobs <- bwlabel(threshImage)

# Step 4: Filter blobs by area (optional)
# Assuming you want to keep blobs around 25 pixels in area
blobAreas <- table(blobs)
blobsFiltered <- blobs %in% names(which(blobAreas >= 20 & blobAreas <= 30))
finalBlobs <- blobs * blobsFiltered

# Display the original and processed images
display(image, method = "raster")
display(finalBlobs, method = "raster")
```

Make sure to replace `'path/to/your/image.jpg'` with the actual path to your image file. The threshold value and the area filter range (`blobAreas >= 20 & blobAreas <= 30`) might need to be adjusted based on the specific characteristics of your image and blobs.

This code provides a basic framework, but you may need to tweak parameters like the threshold and area filter range for optimal results with your specific image.

What does w and h mean? 5

In the context of the `thresh` function from the `EBImage` package in R, the parameters `w` and `h` refer to the width and height, respectively, of the moving window used for local adaptive thresholding. Here's a bit more detail:

1. `w` (Width):
   - This parameter specifies the width of the rectangular window that moves across the image.
   - The algorithm computes a local threshold for each position of this window.
2. `h` (Height):
   - Similarly, this parameter specifies the height of the rectangular window.
   - The local threshold at each position is calculated based on the pixel values within this window.

In local adaptive thresholding, instead of using a global threshold value for the entire image, the algorithm computes different threshold values for different small regions (windows) of the image. This method is particularly effective when different parts of the image have varying lighting conditions or background intensities.

The size of the window (defined by `w` and `h`) is crucial. A window that is too small might be too sensitive to noise, while a window that is too large might not capture the local variations effectively. The optimal size often depends on the specific characteristics of the image and the objects you are trying to segment.

Do you know adaptive optics flood illumination images? 6

Yes, I'm familiar with the concept of adaptive optics in the context of flood illumination imaging. Here's a brief overview:

Adaptive Optics

Adaptive optics (AO) is a technology used to improve the performance of optical systems by reducing the effect of incoming wavefront distortions. It's particularly important in fields like astronomy and ophthalmology, where it's essential to correct for distortions caused by the atmosphere or the eye's cornea and lens.

Flood Illumination Imaging

Flood illumination imaging is a technique where a wide field of light (flood illumination) is used to illuminate the subject. Unlike point or line scanning methods, this technique illuminates the entire field of view at once.

Adaptive Optics in Flood Illumination Imaging

In the context of flood illumination imaging, adaptive optics is used to correct aberrations or distortions across the entire field of view. This is particularly useful in applications like retinal imaging in ophthalmology, where detailed and high-resolution images of the retina are necessary. AO helps in correcting distortions caused by the eye's optics to provide clearer, sharper images.

Applications

- **Astronomy:** For correcting atmospheric distortions to get clearer images of celestial bodies.
- **Ophthalmology:** For high-resolution retinal imaging, which can aid in the diagnosis and monitoring of various eye diseases.

Adaptive optics in flood illumination imaging represents a significant advancement in these fields, allowing for more accurate observations and diagnoses by providing clearer and more detailed images.

I want to perform blob detection analysis on those images 7

Performing blob detection analysis on adaptive optics flood illumination images, especially in a domain like ophthalmology, can be a powerful tool for identifying and analyzing features of interest, such as microaneurysms, drusen, or other retinal structures. Here's a general approach to perform blob detection analysis on these images:

1. Preprocessing

- **Image Quality Enhancement:** Since adaptive optics images are generally high quality, the need for preprocessing might be minimal. However, some standard practices like contrast enhancement might still be beneficial.
- **Noise Reduction:** Apply noise reduction techniques if necessary to ensure that the blobs detected are indeed features of interest and not artifacts.

2. Blob Detection

- **Thresholding:** Apply a thresholding technique to segment the features from the background. Adaptive thresholding might be particularly useful given the variable nature of biological images.
- **Connected Component Analysis:** Use connected component analysis to identify individual blobs. This can be done using functions like `bwlabel` in R or equivalent functions in other software.
- **Feature Extraction:** Extract features of each detected blob, such as size, shape, and intensity. This information can be crucial for subsequent analysis, especially in medical diagnostics.

3. Postprocessing and Analysis

- **Filtering Based on Size/Shape:** Filter out blobs based on size and shape constraints relevant to your study. For example, in retinal imaging, the size of microaneurysms might be known, which can guide the filtering.
- **Statistical Analysis:** Perform statistical analysis on the features of the detected blobs to draw conclusions or for diagnostic purposes.

4. Visualization

- **Overlay Blob Markers:** Overlay markers on the original image at the location of the detected blobs for visual inspection and validation.
- **Create Output Reports:** Generate reports or summaries of the findings, including visualizations and statistical summaries.

Tools and Libraries

- **R (EBImage, imager):** Use R packages like EBImage or imager for image processing.
- **Python (OpenCV, scikit-image):** Python libraries like OpenCV or scikit-image offer extensive functionality for image processing and blob detection.

Example Code in R:

Here's a basic framework using R. This is a generic example; you'll need to adjust it based on your specific image characteristics and analysis requirements.

R Code kopiëren

```R
library(EBImage)

# Read the image
image <- readImage('path/to/image.jpg')

# Preprocess (if necessary)
image <- enhanceContrast(image)

# Apply thresholding
threshImage <- thresh(image, w=15, h=15, offset=0.1)

# Detect blobs
blobs <- bwlabel(threshImage)

# Analyze the blobs
features <- computeFeatures.shape(blobs)
```

Considerations

- Image Characteristics: The parameters for preprocessing and blob detection need to be tailored based on the specific characteristics of your images.
- Domain Knowledge: In medical image analysis, incorporating domain knowledge is crucial for accurate interpretation of results.
- Validation: It's important to validate your blob detection results, possibly by comparing with manual annotations or using known benchmarks.

Counting the number of blobs 8

Counting the number of blobs in an image using blob detection techniques typically involves a few key steps, particularly if you're using a tool like the `EBImage` package in R. Here's a simplified approach:

Steps for Blob Counting:

1. Preprocess the Image:
   - This might include converting it to grayscale (if it's not already), enhancing contrast, and applying noise reduction techniques.

2. Apply Thresholding:
   - Use an appropriate thresholding method to differentiate the blobs (foreground) from the background.
3. Blob Detection:
   - Perform connected component analysis to label each distinct blob in the image.
4. Count the Blobs:
   - Count the number of unique labels assigned to the blobs, which corresponds to the number of blobs.

Example Code in R:

Here's an example R code snippet using `EBImage` for counting blobs:

R Code kopiëren

```r
# Load the EBImage package
library(EBImage)

# Read the image
image <- readImage('path/to/your/image.jpg') # Replace with your image path

# Convert to grayscale if necessary
image <- channel(image, "gray")

# Enhance contrast (optional)
image <- enhanceContrast(image)

# Threshold the image
threshImage <- thresh(image, w=10, h=10, offset=0.05)

# Detect blobs (connected components)
blobs <- bwlabel(threshImage)

# Count the blobs
numBlobs <- max(blobs)

# Print the number of blobs
print(paste("Number of blobs detected:", numBlobs))
```

In this code:

- Adjust the path `'path/to/your/image.jpg'` to the location of your image.
- The `thresh` function parameters (`w`, `h`, `offset`) might need fine-tuning based on your image's specific characteristics.
- The `numBlobs` variable holds the count of detected blobs.

Remember, the accuracy of blob counting highly depends on the quality of the image preprocessing and the parameters used in the thresholding and labeling steps. Fine-tuning these parameters is often necessary to get accurate results, especially in complex images with varying blob sizes and contrast levels.

Max blobs? 9

The use of `max(blobs)` in the context of the R code for blob counting refers to finding the highest label assigned to the connected components in the image. When the `bwlabel` function in the `EBImage` package is used, it labels each connected component (or blob) in the binary image with a unique number. The background is typically labeled as 0, and each blob gets a sequential integer label.

Here's how it works:

- **Labeling:** `bwlabel` assigns labels starting from 1 to each separate blob.
- **Counting:** The maximum label number (`max(blobs)`) corresponds to the total number of blobs detected in the image.

So, if `bwlabel` labels some blobs as 1, 2, 3, ..., N, then `max(blobs)` will return N, which is the count of the blobs.

It's important to note that this approach assumes that the blobs are well-separated and each has been labeled distinctly. If the blobs are touching or not well-separated, they might be labeled as a single blob, which can affect the accuracy of the count. Therefore, proper preprocessing and parameter tuning are crucial for accurate blob detection and counting.

Write the code to display the thresholded image, and another to display the counted blobs in red on the original image 10

To display the thresholded image and to overlay the counted blobs in red on the original image in R using the `EBImage` package, you can follow these steps:

Display the Thresholded Image

First, let's display the thresholded image:

R Code kopiëren

```
# Load the EBImage package
library(EBImage)

# Read the original image
image <- readImage('path/to/your/image.jpg') # Replace with your image path

# Convert to grayscale if necessary
image <- channel(image, "gray")

# Threshold the image
threshImage <- thresh(image, w=10, h=10, offset=0.05)

# Display the thresholded image
display(threshImage, method = "raster")
```

Display Counted Blobs in Red on the Original Image

Now, to overlay the counted blobs in red on the original image:

R Code kopiëren

```
# Detect blobs (connected components)
blobs <- bwlabel(threshImage)

# Create a color image where blobs are red
colorBlobs <- image
colorBlobs[blobs > 0] <- 1 # Set blobs to red in the red channel

# Combine the original image and the red blobs
combinedImage <- combine(image, colorBlobs)

# Display the original image with blobs highlighted in red
display(combinedImage, method = "raster")
```

In this code:

1. `bwlabel` is used to label the blobs in the thresholded image.
2. We create a copy of the original image in `colorBlobs`.
3. For each pixel where `blobs > 0` (i.e., each blob), we set the value to 1 in the red channel of `colorBlobs`, effectively coloring the blobs red.
4. We then use `combine` to overlay this red blob image on top of the original image, creating `combinedImage`.
5. Finally, we display `combinedImage`.

Please adjust the file path and the parameters as per your specific image and requirements. This code assumes that your original image is either grayscale or color. If it's already a binary image, some steps might need to be adjusted.

Error in UseMethod("display", x) :
 no applicable method for 'display' applied to an object of class "c('Image', 'array', 'structure', 'vector')" 11

The error message you're encountering in R suggests that there's an issue with the `display` function when trying to display an image object. This could be due to a variety of reasons, such as a mismatch in expected data types or issues with the specific method implementation for the `Image` class in `EBImage`. Let's address this issue with an alternative approach.

Instead of using `display`, we can save the processed images to files and then view them using an image viewer. Here's how you can modify the code:

Saving the Thresholded Image

R Code kopiëren

```
# Save the thresholded image
saveImage(threshImage, "thresholdedImage.jpg")
```

This will save the thresholded image as "thresholdedImage.jpg" in your working directory. You can open this file with any standard image viewer.

Saving the Original Image with Counted Blobs in Red

Saving the Original Image with Counted Blobs in Red

R Code kopiëren

```
# Create a color image where blobs are red
colorBlobs <- image # Assuming 'image' is in grayscale, we need to convert it to RGB
if (dim(image)[3] != 3) {
    colorBlobs <- gray2rgb(image)
}
colorBlobs[,,1][blobs > 0] <- 1 # Set blobs to red in the red channel

# Save the image with blobs highlighted
saveImage(colorBlobs, "imageWithBlobs.jpg")
```

This will save the original image with the blobs highlighted in red as "imageWithBlobs.jpg". As before, you can view this image using any image viewer.

Please ensure that you have write permissions in your working directory to save these files. Also, adjust the file paths as necessary for your environment.

Error in saveImage(threshImage, "thresholdedImage.jpg") :
could not find function "saveImage" 12

It seems there was a mistake in the previous suggestion. The `EBImage` package in R uses the `writeImage` function, not `saveImage`, to save images to a file. Let's correct that and use `writeImage` to save your images.

Saving the Thresholded Image

R Code kopiëren

```
# Load the EBImage package
library(EBImage)

# Read the original image
image <- readImage('path/to/your/image.jpg') # Replace with your image path

# Convert to grayscale if necessary
image <- channel(image, "gray")

# Threshold the image
threshImage <- thresh(image, w=10, h=10, offset=0.05)

# Save the thresholded image
writeImage(threshImage, "thresholdedImage.jpg")
```

This code will save the thresholded image to a file named "thresholdedImage.jpg" in your current working directory.

Saving the Original Image with Counted Blobs in Red

R Code kopiëren

```
# Detect blobs (connected components)
blobs <- bwlabel(threshImage)

# Create a color image where blobs are red
colorBlobs <- image # Assuming 'image' is in grayscale, we need to convert it to RGB
if (dim(image)[3] != 3) {
    colorBlobs <- gray2rgb(image)
}
colorBlobs[,,1][blobs > 0] <- 1 # Set blobs to red in the red channel

# Save the image with blobs highlighted
writeImage(colorBlobs, "imageWithBlobs.jpg")
```

This code will save the original image with the blobs highlighted in red as "imageWithBlobs.jpg" in your current working directory.

Remember to replace ``'path/to/your/image.jpg'`` with the actual path to your image file. Also, ensure that you have the necessary permissions to write files to the directory where you're running this code.

where does it sav (13)

In R, when you use the `writeImage` function from the `EBImage` package to save an image, the file is saved to the current working directory unless you specify a different path. The current working directory is the folder where your R session is running.

You can find out what your current working directory is in R with the `getwd()` function. If you want to save the image to a specific directory, you need to provide the full path in the `writeImage` function.

Here's how you can check and set your working directory:

1. **Check Current Working Directory:**

R Code kopiëren

```r
getwd()
```

2. Set a New Working Directory (if you want to save files somewhere specific):

R Code kopiëren

```r
setwd('path/to/desired/directory')  # Replace with the desired path
```

3. Save the Image:

R Code kopiëren

```r
writeImage(threshImage, "thresholdedImage.jpg")
```

This will save the image as "thresholdedImage.jpg" in the current working directory.

If you want to save the image directly to a specific location, provide the full path in the `writeImage` call, like this:

R Code kopiëren

```r
writeImage(threshImage, 'path/to/desired/directory/thresholdedImage.jpg')
```

Replace `'path/to/desired/directory/'` with the actual path where you want to save the image. This way, you can directly control where the image file is saved without changing the entire R session's working directory.

+ }
Error in if (dim(image)[3] != 3) { :
 missing value where TRUE/FALSE needed
> colorBlobs[,,1][blobs > 0] <- 1 # Set blobs to red in the red channel
Error in slot(`*tmp*`, ".Data")[, , 1, drop = FALSE] :
 incorrect number of dimensions

(14)

The error you're encountering indicates a problem with the dimensionality of the image being processed. It seems the image may not have the expected three dimensions (height, width, color channels) required for the operation. Let's correct the code to handle this.

Here's an updated version of the script to ensure it works with both grayscale and color images:

Saving the Original Image with Counted Blobs in Red

R   Code kopiëren

```
# Load the EBImage package
library(EBImage)

# Read the original image
image <- readImage('path/to/your/image.jpg') # Replace with your image path

# Convert to grayscale if necessary
grayImage <- channel(image, "gray")

# Threshold the image
threshImage <- thresh(grayImage, w=10, h=10, offset=0.05)

# Detect blobs (connected components)
blobs <- bwlabel(threshImage)

# Check if the original image is grayscale or color
if (length(dim(image)) == 2) {  # Grayscale image
    colorImage <- gray2rgb(image)
} else {  # Color image
    colorImage <- image
}

# Create a mask where blobs are red
redMask <- array(0, dim = dim(colorImage))
redMask[,,1][blobs > 0] <- 1

# Combine the original image and the red mask
combinedImage <- colorImage
combinedImage[redMask > 0] <- 1

# Save the image with blobs highlighted
writeImage(combinedImage, "imageWithBlobs.jpg")
```

This code first checks if the original image is grayscale or already in color. If it's grayscale, it converts it to a color image using `gray2rgb`. Then, it creates a red mask for the blobs and overlays this mask on the color image. Finally, the image with the highlighted blobs is saved.

Make sure to replace `'path/to/your/image.jpg'` with the actual path to your image. Also, adjust the thresholding parameters as needed for your specific image.

Error in gray2rgb(image) : could not find function "gray2rgb" (15)

It appears that I made an error regarding the existence of a `gray2rgb` function in the `EBImage` package. My apologies for the confusion. We need to use an alternative method to convert a grayscale image to RGB format in R.

The process involves creating a three-layered RGB image where each layer (red, green, blue) is initially a copy of the grayscale image. Here's the corrected code:

Corrected Script for Highlighting Blobs

R Code kopiëren

```r
# Load the EBImage package
library(EBImage)

# Read the original image
image <- readImage('path/to/your/image.jpg') # Replace with your image path

# Convert to grayscale if necessary
grayImage <- channel(image, "gray")

# Threshold the image
threshImage <- thresh(grayImage, w=10, h=10, offset=0.05)

# Detect blobs (connected components)
blobs <- bwlabel(threshImage)

# Convert grayscale to RGB if the original image is grayscale
if (length(dim(image)) == 2) {
    # Create a blank RGB image with the same dimensions as the grayscale image
    colorImage <- array(0, dim = c(dim(image), 3))

    # Copy the grayscale image into each color channel
    colorImage[,,1] <- image
    colorImage[,,2] <- image
    colorImage[,,3] <- image
} else {
    # Use the original image if it's already in color
    colorImage <- image
}

# Create a mask where blobs are red
redMask <- blobs > 0

# Overlay the red mask on the original image
# Setting red channel to maximum intensity for the blobs
colorImage[redMask, 1] <- 1
colorImage[redMask, 2:3] <- 0 # Setting green and blue channels to 0 for the blobs

# Save the image with blobs highlighted
writeImage(colorImage, "imageWithBlobs.jpg")
```

In this script:

1. If the original image is grayscale, it creates a blank RGB image and then duplicates the grayscale image into each color channel.
2. A mask is created for the blobs, and this mask is used to set the corresponding pixels in the red channel to maximum intensity (and green/blue to zero) to highlight them in red.
3. Finally, the modified image is saved.

Remember to replace `'path/to/your/image.jpg'` with the actual path to your image file. The thresholding parameters (`w`, `h`, `offset`) might need adjustments based on your specific image's characteristics.

Overlay the red mask on the original image
Setting red channel to maximum intensity for the blobs
colorImage[redMask, 1] <- 1 16
colorImage[redMask, 2:3] <- 0 # Setting green and blue channels to 0 for the blobs

The code snippet you've highlighted is intended to overlay the detected blobs in red on the original image. It modifies the red, green, and blue channels of the color image based on where the blobs are detected. Let's break it down for clarity:

R Code kopiëren

```r
# Overlay the red mask on the original image
# Setting red channel to maximum intensity for the blobs
colorImage[redMask, 1] <- 1
colorImage[redMask, 2:3] <- 0 # Setting green and blue channels to 0 for the blobs
```

Here's what each line does:

1. `colorImage[redMask, 1] <- 1`: This line sets the red channel to maximum intensity (1) for all pixels where `redMask` is `TRUE` (i.e., where blobs are detected). In an RGB image, setting only the red channel to maximum intensity while keeping other channels at minimum (0) makes the pixels appear

> colorImage[redMask, 1] <- 1
Error in colorImage[redMask, 1] <- 1 :
  incorrect number of subscripts on matrix
> colorImage[redMask, 2:3] <- 0 # Setting green and blue channels to 0 for the blobs 17
Error in colorImage[redMask, 2:3] <- 0 :
  incorrect number of subscripts on matrix

The errors you're encountering indicate an issue with the way the subscripting is done for the `colorImage` matrix. In R, when dealing with multi-dimensional arrays (like images), you need to be careful with indexing. Let's correct the code to properly overlay the red mask on the original image.

Here's the corrected approach:

Corrected Script for Overlaying Red Mask

R Code kopiëren

```r
# Assuming 'blobs' is already computed

# Convert grayscale to RGB if the original image is grayscale
if (length(dim(image)) == 2) {
    # Create a blank RGB image with the same dimensions as the grayscale image
    colorImage <- array(0, dim = c(dim(image), 3))

    # Copy the grayscale image into each color channel
    for (i in 1:3) {
        colorImage[,,i] <- image
    }
} else {
    # Use the original image if it's already in color
    colorImage <- image
}

# Create a mask for the blobs
redMask <- blobs > 0

# Overlay the red mask on the original image
colorImage[,,1][redMask] <- 1   # Red channel
colorImage[,,2][redMask] <- 0   # Green channel
colorImage[,,3][redMask] <- 0   # Blue channel

# Save the image with blobs highlighted in red
writeImage(colorImage, "imageWithBlobs.jpg")
```

In this corrected version:

1. We first check if the image is grayscale and convert it to an RGB image if necessary.
2. The `redMask` is used to identify the pixels corresponding to the blobs.
3. We then update the red, green, and blue channels of these pixels. Specifically, we set the red channel to maximum (1) and the green and blue channels to minimum (0) for the pixels where blobs are detected, making them appear red.
4. Finally, the modified image is saved.

This approach should resolve the subscripting issue and properly overlay the blobs in red on the original image. Remember to replace ``'path/to/your/image.jpg'`` with the actual path to your image file.

I get three images without red blobs (18)

If the resulting image doesn't show the red blobs as expected, it's possible that the way we're applying the red mask isn't correctly targeting the blob areas, or there might be issues with the way the blobs were detected initially. Let's troubleshoot this step by step.

Step 1: Verify Blob Detection

First, let's ensure that the blob detection is working correctly:

R Code kopiëren

```R
library(EBImage)

# Read the original image
image <- readImage('path/to/your/image.jpg') # Replace with your image path

# Convert to grayscale if necessary
grayImage <- channel(image, "gray")

# Threshold the image
threshImage <- thresh(grayImage, w=10, h=10, offset=0.05)

# Detect blobs (connected components)
blobs <- bwlabel(threshImage)

# Display the labeled blobs
display(blobs)
```

This will display the image with labeled blobs. Each blob should be distinctly labeled. If this image doesn't show distinct blobs, you may need to adjust the thresholding parameters.

Step 2: Apply the Red Mask Correctly

If blob detection is working as expected, let's apply the red mask correctly:

R Code kopiëren

```r
# Convert grayscale to RGB if the original image is grayscale
if (length(dim(image)) == 2) {
    colorImage <- stack(image, image, image)  # Stacking the grayscale image into three ch
} else {
    colorImage <- image
}

# Create a mask for the blobs
redMask <- blobs > 0

# Overlay the red mask on the original image
colorImage[redMask, 1] <- 1   # Red channel
colorImage[!redMask, 1] <- colorImage[!redMask, 1] # Retain original intensity for non-blo
colorImage[redMask, 2:3] <- 0 # Green and Blue channels

# Save the image with blobs highlighted in red
writeImage(colorImage, "imageWithBlobs.jpg")
```

In this script:

- If the original image is grayscale, it's converted into an RGB image by stacking the grayscale image in three channels.
- The red mask is applied only to the blob areas. The red channel is set to maximum for blobs, while the green and blue channels are set to zero. For non-blob areas, the original intensity is retained in the red channel.

Ensure the path to the image is correctly set. If the blobs still don't appear in red, the issue might be with the blob detection or the specific properties of your image, which may require a different approach or parameter adjustments.

```
if (length(dim(image)) == 2) {
+   colorImage <- stack(image, image, image)  # Stacking the grayscale image into three channels
+ } else {
+   colorImage <- image
+ }
Error in if (drop) { : the condition has length > 1
```

19

The error message indicates a problem with the conditional statement checking the dimensions of the `image`. This issue likely arises because `dim(image)` returns more than one value, and the `if` statement expects a single logical value. To properly check if the image is grayscale (which would have only two dimensions: width and height), we need to modify the condition.

Let's revise the code to handle this correctly:

Corrected Script for Overlaying Red Mask

R Code kopiëren

```r
# Assuming 'blobs' is already computed

# Check if the image is grayscale (2 dimensions) or color (3 dimensions)
if (dim(image)[3] == 1 || length(dim(image)) == 2) {
    # The image is grayscale; convert it to RGB
    colorImage <- abind(image, image, image, along = 3)
} else {
    # The image is already in color
    colorImage <- image
}

# Create a mask for the blobs
redMask <- blobs > 0

# Overlay the red mask on the original image
colorImage[,,1][redMask] <- 1   # Red channel
colorImage[,,2][redMask] <- 0   # Green channel
colorImage[,,3][redMask] <- 0   # Blue channel

# Save the image with blobs highlighted in red
writeImage(colorImage, "imageWithBlobs.jpg")
```

In this script:

1. We check the number of channels in the `image` to determine if it's grayscale or color. For grayscale images, `dim(image)` will return two dimensions, or the third dimension will be 1.
2. If the image is grayscale, we use `abind` from the `abind` package to stack the grayscale image into an RGB format. If you don't have the `abind` package installed, you can install it using `install.packages("abind")`.
3. Then, the red mask is applied only to the detected blobs.
4. Finally, the image with the blobs highlighted in red is saved.

This approach should correctly handle both grayscale and color images. Ensure that the path to the image and the package installations are correctly set up.

max size of a blob is 50 pixels, show me the image of the thresholded image with only elible blobs as well 20

To display and save an image where only the blobs with a maximum size of 50 pixels are shown, we need to modify the script to filter out larger blobs. This involves calculating the area of each blob and then creating a mask to keep only those blobs that are equal to or smaller than the specified size.

Let's modify the script accordingly:

Script to Display and Save Thresholded Image with Blobs up to 50 Pixels

R Code kopiëren

```
# Load necessary libraries
library(EBImage)

# Read the original image
image <- readImage('path/to/your/image.jpg') # Replace with your image path

# Convert to grayscale if necessary
grayImage <- channel(image, "gray")

# Threshold the image
threshImage <- thresh(grayImage, w=10, h=10, offset=0.05)

# Detect blobs (connected components)
blobs <- bwlabel(threshImage)

# Calculate area for each blob
blobAreas <- table(blobs)

# Filter out blobs larger than 50 pixels
eligibleBlobs <- names(blobAreas)[blobAreas <= 50]
eligibleBlobMask <- blobs %in% eligibleBlobs

# Create a new image with only eligible blobs
filteredBlobsImage <- threshImage * eligibleBlobMask

# Display the thresholded image with filtered blobs
display(filteredBlobsImage)

# Save the thresholded image with filtered blobs
writeImage(filteredBlobsImage, 'filteredBlobsImage.jpg')
```

In this code:

1. After detecting the blobs, we calculate the area of each blob.
2. We then create a list of blob labels (`eligibleBlobs`) that have an area of 50 pixels or less.
3. We use this list to create a mask (`eligibleBlobMask`) that will filter out larger blobs.

4. We apply this mask to the thresholded image, resulting in an image that only shows eligible blobs.
5. This filtered image is then displayed and saved.

Remember to replace `'path/to/your/image.jpg'` with the actual path to your image. Also, adjust the thresholding parameters (`w`, `h`, `offset`) as needed for your specific image's characteristics. This script assumes that your blobs are relatively well-separated and that the `bwlabel` function correctly labels each blob.

overlay the filtered blobs in red on the original images and save it using writeimage (21)

To overlay the filtered blobs in red on the original image and save the result, we need to first create a mask based on the filtered blobs and then overlay this mask onto the original image. The blobs will be highlighted in red, while the rest of the image remains unchanged. Here's the script to achieve this:

Script to Overlay Filtered Blobs in Red on Original Image

R Code kopiëren

```r
# Load necessary libraries
library(EBImage)

# Read the original image
image <- readImage('path/to/your/image.jpg') # Replace with your image path

# Convert to grayscale if necessary
grayImage <- channel(image, "gray")

# Threshold the image
threshImage <- thresh(grayImage, w=10, h=10, offset=0.05)

# Detect blobs (connected components)
blobs <- bwlabel(threshImage)

# Calculate area for each blob
blobAreas <- table(blobs)

# Filter out blobs larger than 50 pixels
eligibleBlobs <- names(blobAreas)[blobAreas <= 50]
eligibleBlobMask <- blobs %in% eligibleBlobs

# Convert the original image to RGB if it's grayscale
if (length(dim(image)) == 2) {
    colorImage <- abind(image, image, image, along = 3)
} else {
    colorImage <- image
}
```

```
# Create a mask for filtered blobs
filteredBlobsRedMask <- array(0, dim = dim(colorImage))
filteredBlobsRedMask[,,1][eligibleBlobMask] <- 1 # Red channel

# Overlay the red mask on the original image
colorImage[filteredBlobsRedMask > 0] <- 1

# Save the image with filtered blobs highlighted in red
writeImage(colorImage, 'imageWithFilteredBlobs.jpg')
```

This script:

1. Detects blobs in the thresholded image.
2. Filters out blobs larger than 50 pixels.
3. Converts the original image to an RGB format if it's grayscale.
4. Creates a red mask for the filtered blobs.
5. Overlays this red mask onto the original image, highlighting the filtered blobs in red.
6. Saves the resulting image.

Ensure that the path to the image is correctly set and that you have the `abind` package installed if your image is initially grayscale. Adjust the parameters as needed for your specific image's characteristics.

at threshold 100 by 100 it still misses some blobs 22

If the current thresholding parameters (e.g., `w=100`, `h=100`) are missing some blobs, it suggests that the thresholding isn't adequately capturing the variation in the image, possibly due to the size of the window being too large or other factors like image contrast. Let's explore some strategies to improve blob detection:

1. Adjusting Threshold Parameters

- **Window Size (`w`, `h`)**: Try smaller window sizes. Large windows might average out the local variations too much, especially for small blobs.
- **Offset**: Adjust the offset parameter. This value shifts the threshold level up or down and can be critical for capturing fainter or smaller blobs.

2. Contrast Enhancement

- If the blobs are not distinct enough from the background, enhancing the contrast of the image before thresholding might help.

3. Different Thresholding Methods

- Consider experimenting with different thresholding methods if available. For example, global thresholding or other adaptive methods might yield better results.

Updated Script with Adjustments

R Code kopiëren

```r
# Load necessary libraries
library(EBImage)

# Read the original image
image <- readImage('path/to/your/image.jpg') # Replace with your image path

# Convert to grayscale if necessary
grayImage <- channel(image, "gray")

# Enhance contrast (optional, adjust as needed)
enhancedImage <- enhanceContrast(grayImage, 1.5)  # Adjust the factor as needed

# Adaptive thresholding with smaller window size
threshImage <- thresh(enhancedImage, w=30, h=30, offset=0.1)  # Adjust as needed

# Blob detection and filtering
blobs <- bwlabel(threshImage)
blobAreas <- table(blobs)
eligibleBlobs <- names(blobAreas)[blobAreas <= 50]
eligibleBlobMask <- blobs %in% eligibleBlobs

# Convert the original image to RGB if it's grayscale
if (length(dim(image)) == 2) {
    colorImage <- abind(image, image, image, along = 3)
} else {
    colorImage <- image
}

# Create a mask for filtered blobs
filteredBlobsRedMask <- array(0, dim = dim(colorImage))
filteredBlobsRedMask[,,1][eligibleBlobMask] <- 1 # Red channel

# Overlay the red mask on the original image
colorImage[filteredBlobsRedMask > 0] <- 1

# Save the image with filtered blobs highlighted in red
writeImage(colorImage, 'imageWithFilteredBlobs.jpg')
```

In this script, we added an optional contrast enhancement step and suggested using a smaller window size for the `thresh` function. Adjust these parameters according to the specific characteristics of your image and the blobs you're trying to detect. Fine-tuning these parameters is often crucial for effective blob detection.

rror in enhanceContrast(grayImage, 1.5) :
could not find function "enhanceContrast" (23)

It appears that the `enhanceContrast` function is not available or not correctly referenced in your R environment. My apologies for the oversight. The `EBImage` package provides several other methods for contrast adjustment that can be used instead, such as `normalize` or `equalize`. Let's revise the script using an alternative approach for contrast enhancement:

Revised Script with Contrast Adjustment

R Code kopiëren

```r
# Load necessary libraries
library(EBImage)

# Read the original image
image <- readImage('path/to/your/image.jpg') # Replace with your image path

# Convert to grayscale if necessary
grayImage <- channel(image, "gray")

# Enhance contrast using histogram equalization (alternative approach)
enhancedImage <- equalize(grayImage)

# Adaptive thresholding with adjusted window size
threshImage <- thresh(enhancedImage, w=30, h=30, offset=0.1)  # Adjust as needed

# Blob detection and filtering
blobs <- bwlabel(threshImage)
blobAreas <- table(blobs)
eligibleBlobs <- names(blobAreas)[blobAreas <= 50]
eligibleBlobMask <- blobs %in% eligibleBlobs

# Convert the original image to RGB if it's grayscale
if (length(dim(image)) == 2) {
    colorImage <- abind(image, image, image, along = 3)
} else {
    colorImage <- image
}

# Create a mask for filtered blobs
filteredBlobsRedMask <- array(0, dim = dim(colorImage))
filteredBlobsRedMask[,,1][eligibleBlobMask] <- 1 # Red channel

# Overlay the red mask on the original image
colorImage[filteredBlobsRedMask > 0] <- 1
```

```
# Save the image with filtered blobs highlighted in red
writeImage(colorImage, 'imageWithFilteredBlobs.jpg')
```

In this script, we use `equalize` for contrast enhancement, which applies histogram equalization to the grayscale image. Histogram equalization can improve the contrast of an image by effectively spreading out the most frequent intensity values, which might help in making the blobs more distinguishable for thresholding and detection.

Remember to adjust the window size (`w`, `h`) and the offset in the `thresh` function as needed for your specific image. The effectiveness of this approach depends on the characteristics of the blobs and the background in your image.

blobs larger than 20 pixels (24)

To filter and highlight blobs larger than 20 pixels in an image, you need to modify the blob filtering step in the script. Instead of keeping blobs that are equal to or smaller than a certain size, you'll now keep those that are larger than 20 pixels. Here's the updated script for this purpose:

Script for Filtering and Highlighting Blobs Larger Than 20 Pixels

R Code kopiëren

```
# Load necessary libraries
library(EBImage)

# Read the original image
image <- readImage('path/to/your/image.jpg') # Replace with your image path

# Convert to grayscale if necessary
grayImage <- channel(image, "
```

between 20 and 50 pixels (25)

To filter and highlight blobs with sizes between 20 and 50 pixels, you will need to adjust the blob filtering criteria in the script. This involves modifying the condition to include only blobs within this specific size range. Here's the revised script:

Script for Filtering and Highlighting Blobs Between 20 and 50 Pixels

R Code kopiëren

```
# Load necessary libraries
library(EBImage)

# Read the original image
image <- readImage('path/to/your/image.jpg') # Replace with your image path

# Convert to grayscale if necessary
grayImage <- channel(image, "gray")

# Adaptive thresholding
threshImage <- thresh(grayImage, w=10, h=10, offset=0.05)

# Detect blobs (connected components)
blobs <- bwlabel(threshImage)

# Calculate area for each blob
blobAreas <- table(blobs)

# Filter blobs with size between 20 and 50 pixels
eligibleBlobs <- names(blobAreas)[blobAreas > 20 & blobAreas <= 50]
eligibleBlobMask <- blobs %in% eligibleBlobs

# Convert the original image to RGB if it's grayscale
if (length(dim(image)) == 2) {
    colorImage <- abind(image, image, image, along = 3)
} else {
    colorImage <- image
}

# Create a mask for filtered blobs
filteredBlobsRedMask <- array(0, dim = dim(colorImage))
filteredBlobsRedMask[,,1][eligibleBlobMask] <- 1 # Red channel

# Overlay the red mask on the original image
colorImage[filteredBlobsRedMask > 0] <- 1

# Save the image with filtered blobs highlighted in red
writeImage(colorImage, 'imageWithFilteredBlobs.jpg')
```

In this script:

1. We calculate the area for each blob detected in the thresholded image.
2. We filter out blobs to include only those with sizes between 20 and 50 pixels.
3. We then create a red mask for these filtered blobs and overlay this mask onto the original image.
4. The resulting image, with the specified blobs highlighted in red, is saved.

Please ensure that the path to your image is correctly specified and adjust the thresholding parameters (`w`, `h`, `offset`) based on your image's characteristics. The `abind` function is used to stack grayscale images into RGB format, so make sure you have this function available or adjust the script accordingly for your setup.

I want to capture fainter blobs, should I raise or lower offset 26

To capture fainter blobs in your image processing task, you typically want to **lower the offset** value in the thresholding function. The offset parameter in the `thresh` function adjusts the threshold level, and lowering it can make the algorithm more sensitive to lighter or fainter features in the image.

Here's a brief explanation:

- **Lowering the Offset:** Decreasing the offset value reduces the threshold level, meaning that more pixels will be considered as foreground (potential blobs). This is useful when trying to detect fainter or less pronounced blobs.
- **Raising the Offset:** Increasing the offset value raises the threshold level, causing fewer pixels to be considered as foreground. This is useful for ignoring noise or very faint features that are not of interest.

Adjusted Script for Fainter Blobs

In your script, you would adjust the `offset` parameter in the `thresh` function call:

```R
# Adaptive thresholding with a lower offset
threshImage <- thresh(grayImage, w=10, h=10, offset=0.01)  # Lowered offset
```

In this adjusted line, the `offset` is set to `0.01`, which is lower than the previous value. You might need to experiment with this value to find the right balance for your specific images. Too low of an offset might increase the noise, while too high might miss the faint blobs.

Remember, the effectiveness of these adjustments can vary depending on the specific characteristics of your images, such as contrast, noise level, and the nature of the blobs. Fine-tuning these parameters is often an iterative process.

some blobs are confluent and thereby not captured by the max size, i want to count them 27

When dealing with confluent blobs that are not being accurately captured due to their combined size exceeding the maximum size threshold, you'll need a more sophisticated approach to distinguish and count them. This situation often requires a combination of image processing techniques including more advanced segmentation and possibly morphological operations. Here's a general approach you can take:

1. Preprocess the Image

- Enhance contrast if necessary to make blobs more distinct.
- Apply noise reduction techniques to reduce the chance of false detections.

2. Initial Blob Detection

- Use adaptive thresholding to detect blobs.
- Perform initial blob labeling.

3. Morphological Operations

- **Erosion:** Apply an erosion operation to separate blobs that are close together.
- **Dilation:** Following erosion, dilation can be used to restore the original size of the blobs.

4. Blob Detection on Processed Image

- Redo the blob detection on the morphologically processed image.
- Filter blobs based on size, if necessary.

5. Count the Blobs

Example R Code

R Code kopiëren

```r
library(EBImage)

# Read and preprocess the image
image <- readImage('path/to/your/image.jpg')
grayImage <- channel(image, "gray")
enhancedImage <- equalize(grayImage)

# Thresholding
threshImage <- thresh(enhancedImage, w=10, h=10, offset=0.05)

# Morphological operations
erodedImage <- erode(threshImage, makeBrush(5, shape='diamond'))  # Adjust brush size as n
dilatedImage <- dilate(erodedImage, makeBrush(5, shape='diamond'))

# Blob detection on processed image
processedBlobs <- bwlabel(dilatedImage)

# Counting the blobs
blobCount <- max(processedBlobs)

# Display blob count
print(paste("Number of blobs detected:", blobCount))
```

Notes:

- **Morphological Operations:** The `erode` and `dilate` functions are used here for morphological operations. The `makeBrush` function creates a structuring element, whose size and shape might need to be adjusted based on the specifics of your blobs.
- **Blob Counting:** The `max(processedBlobs)` gives the count of the blobs.
- **Parameter Tuning:** The success of this approach relies heavily on correctly tuning the parameters for thresholding and morphological operations based on the specific characteristics of your image and the blobs.

This approach is a starting point and might require iterative adjustments. Morphological operations can help in separating blobs that are touching or nearly touching, but finding the right balance between erosion and dilation is key to accurately counting the blobs.

> erodedImage <- erode(threshImage, makeBrush(5, shape='diamond')) # Adjust brush size as needed
Error: Expecting a four-dimensional array 28
> dilatedImage <- dilate(erodedImage, makeBrush(5, shape='diamond'))
Error: object 'erodedImage' not found

The error you're encountering with the `erode` function in R's `EBImage` package is likely due to the input image not being in the expected format. The `erode` function requires the image to be a binary image (i.e., an image with two dimensions where the pixel values are either 0 or 1). Let's ensure that the image is correctly processed before applying the morphological operations.

Revised Script with Morphological Operations

First, make sure that your image is a binary image after thresholding. Then apply the erosion and dilation:

R Code kopiëren

```r
library(EBImage)

# Read and preprocess the image
image <- readImage('path/to/your/image.jpg') # Replace with your image path
grayImage <- channel(image, "gray")

# Adaptive thresholding
threshImage <- thresh(grayImage, w=10, h=10, offset=0.05)

# Ensure the image is binary for morphological processing
binaryImage <- threshImage > 0
```

```
# Morphological operations
erodedImage <- erode(binaryImage, makeBrush(5, shape='diamond'))  # Adjust brush size as n
dilatedImage <- dilate(erodedImage, makeBrush(5, shape='diamond'))

# Blob detection on processed image
processedBlobs <- bwlabel(dilatedImage)

# Counting the blobs
blobCount <- max(processedBlobs)

# Display blob count
print(paste("Number of blobs detected:", blobCount))
```

In this script:

- After thresholding, the image is converted into a binary format using `binaryImage <- threshImage > 0`. This ensures compatibility with the morphological functions.
- The `erode` and `dilate` functions are then applied to the binary image.
- The brush size in `makeBrush` may need to be adjusted depending on the size and proximity of the blobs in your image.
- Finally, the blobs in the processed image are detected and counted.

Please adjust the file path and the parameters according to your specific needs. Morphological operations can be sensitive to the size of the structuring element (`makeBrush`), so you might need to experiment with different sizes to achieve the best results for your specific image.

> erodedImage <- erode(binaryImage, makeBrush(5, shape='diamond')) # Adjust brush size as needed
> Error: Expecting a four-dimensional array

29

The error message "Expecting a four-dimensional array" suggests that there's a mismatch between the expected input format for the `erode` function and the actual format of the image being provided. In `EBImage`, images are expected to be either grayscale (two-dimensional) or color (three-dimensional). However, the error message indicates that a four-dimensional array is expected, which is unusual for typical image processing tasks in `EBImage`.

Let's troubleshoot this issue:

1. **Verify Image Dimensions:** First, check the dimensions of the `binaryImage` to ensure it's either two-dimensional (for grayscale images) or three-dimensional (for color images).

R

```
print(dim(binaryImage))
```

2. Apply Morphological Operations Correctly: Assuming `binaryImage` is correctly formatted as a two-dimensional array (which it should be after thresholding), try using the `erode` function without specifying the shape of the brush, as the `makeBrush` function might be causing the issue.

Modified Script

R

```
library(EBImage)

# Read and preprocess the image
image <- readImage('path/to/your/image.jpg') # Replace with your image path
grayImage <- channel(image, "gray")

# Adaptive thresholding
threshImage <- thresh(grayImage, w=10, h=10, offset=0.05)

# Convert to binary image
binaryImage <- threshImage > 0

# Check the dimensions of the binaryImage
print(dim(binaryImage))

# Morphological operations without specifying brush shape
erodedImage <- erode(binaryImage, 5)  # Using a disk of radius 5
dilatedImage <- dilate(erodedImage, 5)

# Blob detection on processed image
processedBlobs <- bwlabel(dilatedImage)

# Counting the blobs
blobCount <- max(processedBlobs)

# Display blob count
print(paste("Number of blobs detected:", blobCount))
```

This modified script uses a simpler version of the `erode` and `dilate` functions with a disk-shaped structuring element of radius 5 (the number 5 here is an example and should be adjusted based on your needs).

If you continue to encounter issues, it's possible that there might be a version mismatch or other deeper issues with the `EBImage` package or its dependencies. In that case, reinstalling `EBImage` or consulting its documentation for any version-specific changes might be necessary.

Can I preprocess more except for equalize? On the naked eye I can see distuingish all blobs 30

If you can visually distinguish all the blobs with the naked eye but are facing difficulties in processing them programmatically, it indicates that the preprocessing steps need to be refined to better highlight the features of interest. Here are some additional preprocessing techniques you can try in conjunction with or in place of histogram equalization:

1. Contrast Adjustment

- **Linear Contrast Stretching:** Enhances the contrast by stretching the range of intensity values.
- **Gamma Correction:** Adjusts the overall brightness and can be particularly useful for images with varying lighting conditions.

2. Smoothing and Noise Reduction

- **Gaussian Blur:** Smoothens the image and can help in reducing noise, which might make the blobs more distinct.
- **Median Filtering:** Reduces noise while preserving edges, useful for keeping the shape of the blobs intact.

3. Edge Enhancement

- **Unsharp Masking:** Enhances edges by subtracting a blurred (unsharp) version of the image from the original, improving edge contrast.
- **Sobel Operator:** Emphasizes edges in the image, which might help in separating closely situated blobs.

Updated R Script with Additional Preprocessing

Here's how you can incorporate these techniques into your R script:

R Code kopiëren

```r
library(EBImage)

# Read the original image
image <- readImage('path/to/your/image.jpg') # Replace with your image path
grayImage <- channel(image, "gray")

# Contrast adjustment
adjustedImage <- normalize(grayImage)  # Linear contrast stretching
adjustedImage <- gblur(adjustedImage, sigma=1)  # Gaussian blur for noise reduction

# Edge enhancement (optional, use if it helps in your case)
# adjustedImage <- unsharpMask(adjustedImage, radius=1, amount=1.5)
```

```
# Edge enhancement (optional, use if it helps in your case)
# adjustedImage <- unsharpMask(adjustedImage, radius=1, amount=1.5)

# Thresholding
threshImage <- thresh(adjustedImage, w=10, h=10, offset=0.05)

# ... Rest of the blob detection and processing ...
```

Notes:

- **Experiment with Parameters:** The effectiveness of these techniques depends on the specific characteristics of your image. You may need to experiment with the parameters for each function (like the radius in `gblur` and `unsharpMask`) to find the optimal settings for your images.
- **Order of Operations:** The order in which you apply these preprocessing steps can also impact the results. You might need to experiment with different sequences of these operations.
- **Visual Inspection:** After each preprocessing step, you can use `display()` to visually inspect the effects. This can help you fine-tune the parameters and decide which operations are beneficial for your specific case.

Remember, preprocessing in image processing often requires a bit of trial and error, especially when dealing with complex or varied image data. The goal is to enhance the features of interest (in your case, the blobs) so that they can be easily segmented and analyzed in subsequent steps.

count only blobs with w/h>0.7 31

To count only the blobs where the width-to-height ratio (w/h) is greater than 0.7, you'll need to analyze each blob's dimensions after detecting them. In R, using the `EBImage` package, you can compute features for each blob, including their width and height, and then filter based on the specified ratio.

Here's a step-by-step approach:

1. **Blob Detection:** Use thresholding and connected component labeling to detect blobs.
2. **Feature Computation:** Compute blob features, including dimensions.
3. **Filter Blobs:** Filter out blobs based on the width-to-height ratio condition.
4. **Count Valid Blobs:** Count the blobs that meet the specified criterion.

Example R Code

R Code kopiëren

```r
library(EBImage)

# Read and preprocess the image
image <- readImage('path/to/your/image.jpg') # Replace with your image path
grayImage <- channel(image, "gray")
threshImage <- thresh(grayImage, w=10, h=10, offset=0.05)

# Detect blobs
blobs <- bwlabel(threshImage)

# Compute features of each blob
features <- computeFeatures.shape(blobs)

# Calculate width-to-height ratio for each blob
ratios <- features$width / features$height

# Filter blobs with width-to-height ratio > 0.7
validBlobs <- which(ratios > 0.7)

# Count valid blobs
validBlobCount <- length(validBlobs)

# Display the count
print(paste("Number of blobs with w/h ratio > 0.7:", validBlobCount))
```

Important Points:

- Adjust Preprocessing: Depending on your image characteristics, you might need to adjust the preprocessing steps (like thresholding).
- Feature Computation: `computeFeatures.shape` computes various shape-related features. The `width` and `height` properties can be used to calculate the width-to-height ratio.
- Ratios and Filtering: After computing the ratios, we use logical indexing to find and count blobs that meet the criterion.

Make sure to replace `'path/to/your/image.jpg'` with the correct path to your image. Fine-tuning the thresholding and other preprocessing parameters might be necessary based on the nature of your images and the blobs you're trying to detect and analyze.

Feeding ChatGPT earlier generated code to stay on track

32

```
# Load necessary libraries
library(EBImage)

# Read the original image
image <- readImage('path/to/your/image.jpg') # Replace with your
image path

# Convert to grayscale if necessary
grayImage <- channel(image, "gray")

# Adaptive thresholding
threshImage <- thresh(grayImage, w=10, h=10, offset=0.05)

# Detect blobs (connected components)
blobs <- bwlabel(threshImage)

# Calculate area for each blob
blobAreas <- table(blobs)

# Filter blobs with size between 20 and 50 pixels
eligibleBlobs <- names(blobAreas)[blobAreas > 20 & blobAreas <=
50]
eligibleBlobMask <- blobs %in% eligibleBlobs

# Convert the original image to RGB if it's grayscale
if (length(dim(image)) == 2) {
  colorImage <- abind(image, image, image, along = 3)
} else {
  colorImage <- image
}

# Create a mask for filtered blobs
filteredBlobsRedMask <- array(0, dim = dim(colorImage))
filteredBlobsRedMask[,,1][eligibleBlobMask] <- 1 # Red channel

# Overlay the red mask on the original image
colorImage[filteredBlobsRedMask > 0] <- 1

# Save the image with filtered blobs highlighted in red
writeImage(colorImage, 'imageWithFilteredBlobs.jpg')
 combine with this
```

To combine the requirement of filtering blobs based on both size (between 20 and 50 pixels) and aspect ratio (width-to-height ratio greater than 0.7), you will need to first filter the blobs by size, then compute their shape features, and finally filter them based on the aspect ratio. Let's integrate this into your existing script.

Combined R Script for Blob Filtering and Highlighting

R Code kopiëren

```r
# Load necessary libraries
library(EBImage)

# Read the original image
image <- readImage('path/to/your/image.jpg') # Replace with your image path

# Convert to grayscale if necessary
grayImage <- channel(image, "gray")

# Adaptive thresholding
threshImage <- thresh(grayImage, w=10, h=10, offset=0.05)

# Detect blobs (connected components)
blobs <- bwlabel(threshImage)

# Calculate area for each blob
blobAreas <- table(blobs)

# Filter blobs with size between 20 and 50 pixels
eligibleBlobsBySize <- names(blobAreas)[blobAreas > 20 & blobAreas <= 50]
eligibleBlobMaskBySize <- blobs %in% eligibleBlobsBySize

# Compute features of each blob
features <- computeFeatures.shape(blobs)

# Calculate width-to-height ratio for each blob
ratios <- features$width / features$height

# Filter blobs with width-to-height ratio > 0.7
eligibleBlobsByRatio <- names(which(ratios > 0.7))
eligibleBlobMaskByRatio <- blobs %in% eligibleBlobsByRatio

# Combine both size and ratio criteria
combinedEligibleBlobMask <- eligibleBlobMaskBySize & eligibleBlobMaskByRatio

# Convert the original image to RGB if it's grayscale
if (length(dim(image)) == 2) {
    colorImage <- abind(image, image, image, along = 3)
} else {
    colorImage <- image
}

# Create a mask for filtered blobs
filteredBlobsRedMask <- array(0, dim = dim(colorImage))
filteredBlobsRedMask[,,1][combinedEligibleBlobMask] <- 1 # Red channel

# Overlay the red mask on the original image
colorImage[filteredBlobsRedMask > 0] <- 1

# Save the image with filtered blobs highlighted in red
writeImage(colorImage, 'imageWithFilteredAndRatioCheckedBlobs.jpg')
```

Important Notes:

1. **Size Filtering:** Initially, blobs are filtered based on size (between 20 and 50 pixels).
2. **Aspect Ratio Filtering:** The script then calculates the width-to-height ratio and filters blobs where this ratio is greater than 0.7.
3. **Combining Filters:** The final mask (`combinedEligibleBlobMask`) is a combination of both size and ratio filters.
4. **Visualization and Saving:** The script highlights the filtered blobs on the original image and saves it.

Make sure to replace `'path/to/your/image.jpg'` with the actual path to your image. Adjust the parameters and thresholds as necessary for your specific case. The script assumes that the blobs are distinct enough to be correctly labeled and measured by `computeFeatures.shape`.

Error in features$width : $ operator is invalid for atomic vectors 33

The error "operator is invalid for atomic vectors" suggests that the `features` variable is not a data frame or list as expected, but rather an atomic vector. This can happen if the `computeFeatures.shape` function doesn't return the expected type of output. Let's troubleshoot and correct this.

Troubleshooting Steps:

1. **Inspect the Output of `computeFeatures.shape`:**
   - First, check what the `computeFeatures.shape` function returns for your blobs. This will help us understand the structure of the `features` variable.
2. **Adjust the Script Accordingly:**
   - Depending on the structure of the output, we'll need to modify the script to correctly access the width and height attributes of each blob.

Modified Script with Troubleshooting:

R Code kopiëren

```r
library(EBImage)

# Read the original image and preprocess it
image <- readImage('path/to/your/image.jpg') # Replace with your image path
grayImage <- channel(image, "gray")
threshImage <- thresh(grayImage, w=10, h=10, offset=0.05)
```

```
# Detect blobs
blobs <- bwlabel(threshImage)

# Compute features for each blob
features <- computeFeatures.shape(blobs)

# Inspect the structure of the features
print(str(features))

# Continue with the rest of your processing...
```

After running this modified script, look at the output of `print(str(features))`. This will give you an idea of the structure of the `features` variable. You should see something resembling a data frame or a matrix with named columns, including 'width' and 'height'. If it's not the case, the way to access these attributes will need to be adjusted accordingly.

Continue After Inspection:

Once you understand the structure of `features`, you can then extract the width and height correctly. If `features` is indeed a data frame with 'width' and 'height' columns, the original approach should work. If not, you'll need to adapt the code to fit the actual structure of `features`.

Please proceed with these troubleshooting steps and let me know the output of `print(str(features))`, so I can provide further assistance if needed.

```
num [1:8157, 1:6] 1 12 9 10 9 57 16 1 4 145 ...
 - attr(*, "dimnames")=List of 2
  ..$ : chr [1:8157] "1" "2" "3" "4" ...
  ..$ : chr [1:6] "s.area" "s.perimeter" "s.radius.mean" "s.radius.sd" ...
NULL
```

34

The output you've provided indicates that `features` is a numerical matrix with dimension names, not a data frame. This is why the `$` operator isn't working – it's used for accessing columns in data frames and named lists, not matrices.

Given that `features` is a matrix, you can access the width and height using indexing. However, it seems that `computeFeatures.shape` does not directly provide width and height but rather area, perimeter, and radius-related measurements. If width and height are not directly available, they may need to be estimated from the available measurements.

Here's how you can proceed:

Modified Script for Blob Filtering Based on Size (Area)

Since direct width and height are not available, let's use the area (`s.area`) for filtering:

Modified Script for Blob Filtering Based on Size (Area)

Since direct width and height are not available, let's use the area (`s.area`) for filtering:

R Code kopiëren

```R
library(EBImage)

# Read the original image and preprocess it
image <- readImage('path/to/your/image.jpg') # Replace with your image path
grayImage <- channel(image, "gray")
threshImage <- thresh(grayImage, w=10, h=10, offset=0.05)

# Detect blobs
blobs <- bwlabel(threshImage)

# Compute features for each blob
features <- computeFeatures.shape(blobs)

# Extract the area for each blob (assuming the first column is the area)
blobAreas <- features[, "s.area"]

# Filter blobs by area (e.g., between 20 and 50 pixels)
eligibleBlobMask <- blobAreas > 20 & blobAreas <= 50

# Apply this mask to the blobs
filteredBlobs <- blobs
filteredBlobs[!eligibleBlobMask[blobs]] <- 0

# Proceed with visualization and saving...
```

Note:

- This script assumes that the first column (`s.area`) represents the area of each blob. The filter is applied based on this area.
- Since `features` is a matrix, we use `features[, "s.area"]` to get the area values.
- Adjust the area filter range (20 to 50 pixels) as per your requirements.

If you specifically need to filter based on width-to-height ratio, you'll need a different approach, possibly involving estimating the width and height from the available measurements, which might be complex and less accurate. For most blob analysis tasks, using area as a proxy for size is usually sufficient.

Feeding ChatGPT code to enhance further

35

Combining earlier generated pre-processing steps

Extrapolated the 'writeImage' line

```
# Convert to grayscale if necessary
#image <- channel(image, "gray")

# Enhance contrast (optional)
Eimage <- equalize(image)
FImage <- normalize(image)  # Linear contrast stretching
GImage <- gblur(FImage, sigma=1.1)  # Gaussian blur for noise
reduction

# Threshold the image
threshImage <- thresh(GImage, w=9, h=9, offset=0.093)

# Save the thresholded image
writeImage(threshImage, "thresholdedImage.jpg")
writeImage(Eimage, "EImage.jpg")
writeImage(FImage, "FImage.jpg")
writeImage(GImage, "GImage.jpg")

# Detect blobs (connected components)
blobs <- bwlabel(threshImage)

# Calculate area for each blob
blobAreas <- table(blobs)

# Filter blobs with size between 20 and 50 pixels
eligibleBlobs <- names(blobAreas)[blobAreas > 10 & blobAreas <=
50]
eligibleBlobMask <- blobs %in% eligibleBlobs

# Count the blobs
numBlobs <- max(blobs)

# Print the number of blobs
print(paste("Number of blobs detected:", numBlobs))

# Assuming 'blobs' is already computed


# Convert the original image to RGB if it's grayscale
if (length(dim(image)) == 2) {
  colorImage <- abind(image, image, image, along = 3)
} else {
  colorImage <- image
}
```

# Create a mask for filtered blobs
filteredBlobsRedMask <- array(0, dim = dim(colorImage))
filteredBlobsRedMask[,,1][eligibleBlobMask] <- 1 # Red channel

Overlay the red mask on the original image
colorImage[filteredBlobsRedMask > 0] <- 1

Save the image with filtered blobs highlighted in red
writeImage(colorImage, 'imageWithFilteredBlobs.jpg') I am content with this settings, now I want to do this on a batch of images, with the number of blobs written in the image and a data frame on the end with the values

To process a batch of images and annotate each with the number of detected blobs, followed by creating a data frame with these values, you can set up a loop in R to go through each image. Let's break down the process:

1. **Set Up a Batch Processing Loop:** Loop through each image file in your batch.
2. **Process Each Image:** Apply your image processing steps to each image.
3. **Annotate Images:** Write the number of blobs on each processed image.
4. **Collect Data:** Store the number of blobs for each image in a data frame.

Here's an example script to accomplish this:

R Script for Batch Processing

R Code kopiëren

```r
library(EBImage)
library(abind)

# List of image file paths
image_files <- c('path/to/image1.jpg', 'path/to/image2.jpg', ...)  # Replace with actual p

# Initialize a data frame to store results
results <- data.frame(fileName = character(), blobCount = integer(), stringsAsFactors = FA

# Loop through each image file
for (file in image_files) {
  image <- readImage(file)

  # Apply your image processing steps here
  # ... (use your existing image processing code)

  # Count the blobs
  numBlobs <- max(blobs)

  # Annotate the image with the number of blobs
  colorImage <- annotate(colorImage, paste("Blobs:", numBlobs), xy = c(10, 10), col = "red
```

```
  # Save the processed image
  outputFileName <- paste0('processed_', basename(file))
  writeImage(colorImage, outputFileName)

  # Store the result in the data frame
  results <- rbind(results, data.frame(fileName = basename(file), blobCount = numBlobs))
}

# Print the results
print(results)
```

Important Points:

- Image Files: Replace `image_files` with the actual paths of your images.
- Processing Steps: Insert your specific image processing steps in the place of `# ... (use your existing image processing code)`.
- Annotating Images: The `annotate` function is used to write text on images. Adjust the position (`xy = c(10, 10)`) and color (`col = "red"`) as needed.
- Results Data Frame: The `results` data frame will contain the filename and the corresponding blob count.

Running the Script:

- Ensure all the image paths in `image_files` are correct.
- The script will process each image, save the annotated version, and record the number of blobs.
- After processing, `results` will contain a summary of your analysis.

This script provides a basic framework. Depending on your specific requirements and the setup of your R environment, you might need to adjust the file paths, annotation details, and other parameters.

want to import a folder 36

To process all the images in a folder, you can use R to list all the image files in that folder and then loop through them as described in the previous example. Here's how you can modify the script to process images from a specified folder:

R Script for Processing Images in a Folder

R Code kopiëren

```
library(EBImage)
library(abind)
```

```
# Specify the folder containing the images
folderPath <- 'path/to/your/image/folder'  # Replace with your folder path

# Get a list of image file paths
image_files <- list.files(folderPath, pattern = '\\.(jpg|jpeg|png)$', full.names = TRUE)

# Initialize a data frame to store results
results <- data.frame(fileName = character(), blobCount = integer(), stringsAsFactors = FA

# Loop through each image file
for (file in image_files) {
  image <- readImage(file)

  # Apply your image processing steps here
  # ... (use your existing image processing code)

  # Count the blobs
  numBlobs <- max(blobs)

  # Annotate the image with the number of blobs
  colorImage <- annotate(colorImage, paste("Blobs:", numBlobs), xy = c(10, 10), col = "red

  # Save the processed image
  outputFileName <- paste0(folderPath, '/processed_', basename(file))
  writeImage(colorImage, outputFileName)

  # Store the result in the data frame
  results <- rbind(results, data.frame(fileName = basename(file), blobCount = numBlobs))
}

# Print the results
print(results)
```

Important Points:

- Folder Path: Set `folderPath` to the path of your folder containing the images.
- File Types: The `list.files` function is used to retrieve the file paths of images. The `pattern` argument is set to accept JPEG and PNG files. Adjust this pattern if you have different image formats.
- Results: The script processes each image in the folder, annotates it with the blob count, saves the processed image, and records the results in a data frame.

Run this script after replacing `folderPath` with the actual path to your folder containing the images. This script will process all JPEG and PNG images in the specified folder, annotate them, save the processed versions, and compile the results in a data frame.

Error in annotate(colorImage, paste("Blobs:", numBlobs), xy = c(10, 10),
:
could not find function "annotate" (37)

The error "could not find function 'annotate'" indicates that the `annotate` function is not available in the packages we've loaded. In `EBImage`, the equivalent functionality can be achieved using the `drawText` function to add text annotations to images.

Let's modify the script to use `drawText` instead of `annotate`:

Modified R Script for Processing Images in a Folder

R Code kopiëren

```r
library(EBImage)
library(abind)

# Specify the folder containing the images
folderPath <- 'path/to/your/image/folder'  # Replace with your folder path

# Get a list of image file paths
image_files <- list.files(folderPath, pattern = '\\.(jpg|jpeg|png)$', full.names = TRUE)

# Initialize a data frame to store results
results <- data.frame(fileName = character(), blobCount = integer(), stringsAsFactors = FA

# Loop through each image file
for (file in image_files) {
  image <- readImage(file)

  # Apply your image processing steps here
  # ... (use your existing image processing code)

  # Count the blobs
  numBlobs <- max(blobs)

  # Convert the original image to RGB if it's grayscale for annotation
  if (length(dim(image)) == 2) {
    annotatedImage <- abind(image, image, image, along = 3)
  } else {
    annotatedImage <- image
  }

  # Annotate the image with the number of blobs
  annotatedImage <- drawText(annotatedImage, paste("Blobs:", numBlobs), x = 10, y = 10, co

  # Save the processed image
  outputFileName <- paste0(folderPath, '/processed_', basename(file))
  writeImage(annotatedImage, outputFileName)
```

```
  # Store the result in the data frame
  results <- rbind(results, data.frame(fileName = basename(file), blobCount = numBlobs))
}

# Print the results
print(results)
```

Important Points:

- Text Annotation: We use `drawText` to annotate the image with the number of blobs. Adjust the `x`, `y`, `col`, and `size` parameters as needed for the text position, color, and size.
- Image Conversion: Before annotating, the script checks if the image is grayscale and converts it to RGB to ensure that the text color (red) is correctly applied.
- Results: The script processes each image, annotates it with the blob count, saves the annotated version, and stores the results in a data frame.

Replace `folderPath` with the path to your folder and run the script. This will process all images in the specified folder, annotate them with the number of detected blobs, and compile the results in a data frame.

Make voronoi based on blobs 38

Creating a Voronoi diagram based on the positions of detected blobs in an image is a two-step process. First, you need to identify the centroids of the blobs, and then use these points as seeds to generate the Voronoi diagram. In R, this can be done using packages like `EBImage` for blob detection and `spatstat` or similar for creating the Voronoi diagram.

Here's an outline of how you can do it:

1. Blob Detection and Centroid Calculation

Detect the blobs and calculate their centroids using the `computeFeatures.moment` function from `EBImage`.

2. Create Voronoi Diagram

Use the centroids to create a Voronoi diagram. The `spatstat` package is useful for spatial analysis and can create Voronoi diagrams.

Example R Code

First, ensure you have the necessary packages:

R Code kopiëren

```r
if (!requireNamespace("EBImage", quietly = TRUE))
    install.packages("BiocManager")
BiocManager::install("EBImage")

if (!requireNamespace("spatstat", quietly = TRUE))
    install.packages("spatstat")
```

Now, let's proceed with the code:

R Code kopiëren

```r
library(EBImage)
library(spatstat)

# Read and process the image
image <- readImage('path/to/your/image.jpg') # Replace with your image path
grayImage <- channel(image, "gray")
threshImage <- thresh(grayImage, w=10, h=10, offset=0.05)
blobs <- bwlabel(threshImage)

# Compute centroids of blobs
blobFeatures <- computeFeatures.moment(blobs)
centroids <- cbind(blobFeatures$m.cx, blobFeatures$m.cy)

# Convert centroids to a spatial point pattern
window <- owin(xrange = c(0, dim(image)[2]), yrange = c(0, dim(image)[1]))
pointsPattern <- ppp(centroids[,1], centroids[,2], window = window)

# Create Voronoi tessellation
voronoiDiagram <- dirichlet(pointsPattern)

# Plot Voronoi diagram
plot(voronoiDiagram)
```

Notes:

- Blob Detection: This script uses your existing approach for blob detection.
- Centroids: The centroids of the blobs are calculated as potential seeds for the Voronoi diagram.
- Voronoi Diagram: The `spatstat` package is used to create and plot the Voronoi diagram based on these centroids.

Adjustments and Considerations:

- Ensure that the image path is correctly set.
- The thresholding and blob detection parameters may need adjustment depending on your specific image characteristics.

- The Voronoi diagram is plotted in a coordinate space defined by the image dimensions. If the image is large, you might want to resize or adjust the plot settings for better visualization.

This script provides a basic framework for generating a Voronoi diagram based on blob centroids. Depending on your exact requirements and the nature of your images, further adjustments might be necessary.

Feeding earlier generated code to stay on track

39

```
# Read the image
image <-
readImage('E:/V2/
1.png') # Replace with your image path

# Convert to grayscale if necessary
#image <- channel(image, "gray")

# Enhance contrast (optional)
Eimage <- equalize(image)
FImage <- normalize(image)  # Linear contrast stretching
GImage <- gblur(FImage, sigma=1.1)  # Gaussian blur for noise
reduction

# Threshold the image
threshImage <- thresh(GImage, w=7, h=7, offset=0.035)

# Save the thresholded image
writeImage(threshImage, "thresholdedImage.jpg")
writeImage(Eimage, "EImage.jpg")
writeImage(FImage, "FImage.jpg")
writeImage(GImage, "GImage.jpg")

# Detect blobs (connected components)
blobs <- bwlabel(threshImage)

# Calculate area for each blob
blobAreas <- table(blobs)

# Filter blobs with size between 20 and 50 pixels
eligibleBlobs <- names(blobAreas)[blobAreas > 10 & blobAreas <=
80]
eligibleBlobMask <- blobs %in% eligibleBlobs

# Count the blobs
numBlobs <- max(blobs)

count <- sum(blobAreas > 10 & blobAreas < 80)

# Print the result
print(paste("Number of elements above 10 and below 80:", count))
```

# Print the number of blobs
print(paste("Number of blobs detected:", numBlobs))

Assuming 'blobs' is already computed

# Convert the original image to RGB if it's grayscale
if (length(dim(image)) == 2) {
  colorImage <- abind(image, image, image, along = 3)
} else {
  colorImage <- image
}

# Create a mask for filtered blobs
filteredBlobsRedMask <- array(0, dim = dim(colorImage))
filteredBlobsRedMask[,,1][eligibleBlobMask] <- 1 # Red channel

Overlay the red mask on the original image
colorImage[filteredBlobsRedMask > 0] <- 1

Save the image with filtered blobs highlighted in red
writeImage(colorImage, 'imageWithFilteredBlobs.jpg') I want to perform watershed analysis after the thresholding, and writeImage the result after it

To perform a watershed analysis after thresholding in `EBImage`, you can use the `watershed` function. This function is designed to segment the image into regions based on the gradient of the image, which is particularly useful for separating touching or overlapping objects that might be considered a single blob after thresholding.

Here's how you can integrate watershed segmentation into your script and then save the result:

Modified Script with Watershed Analysis

R

Code kopiëren

```
library(EBImage)
# library(abind) # Not needed for the modified script

# Read the image
image <- readImage('E:[redacted].png')

# Apply preprocessing steps
Eimage <- equalize(image)
FImage <- normalize(image)  # Linear contrast stretching
GImage <- gblur(FImage, sigma=1.1)  # Gaussian blur for noise reduction

# Threshold the image
threshImage <- thresh(GImage, w=7, h=7, offset=0.035)

# Perform watershed analysis on the thresholded image
# It's often useful to invert the thresholded image for the watershed analysis,
# as the watershed algorithm treats high-intensity regions as barriers
wsResult <- watershed(-threshImage, mask = threshImage > 0)

# Save the thresholded image and preprocessing results
writeImage(threshImage, "thresholdedImage.jpg")
writeImage(Eimage, "EImage.jpg")
writeImage(FImage, "FImage.jpg")
writeImage(GImage, "GImage.jpg")

# Save the watershed result
writeImage(wsResult, 'watershedResult.jpg')

# Detect blobs (connected components) in the watershed result
blobs <- bwlabel(wsResult > 0)

# Calculate area for each blob
blobAreas <- table(blobs)

# Filter blobs with size between 20 and 80 pixels
eligibleBlobs <- names(blobAreas)[blobAreas > 10 & blobAreas <= 80]
eligibleBlobMask <- blobs %in% eligibleBlobs

# Count the filtered blobs
numBlobs <- sum(blobAreas > 10 & blobAreas <= 80)

# Print the result
print(paste("Number of filtered blobs:", numBlobs))

# Convert the original image to RGB if it's grayscale
if (length(dim(image)) == 2) {
```

```
} else {
  colorImage <- image
}

# Create a mask for filtered blobs
filteredBlobsRedMask <- array(0, dim = dim(colorImage))
filteredBlobsRedMask[,,1][eligibleBlobMask] <- 1 # Red channel

# Overlay the red mask on the original image
colorImage[filteredBlobsRedMask > 0] <- 1

# Save the image with filtered blobs highlighted in red
writeImage(colorImage, 'imageWithFilteredBlobs.jpg')
```

Notes:

- **Watershed Analysis:** The `watershed` function is applied to the negative of the thresholded image (`-threshImage`), using the thresholded image itself as a mask (`mask = threshImage > 0`). This approach helps in segmenting the regions between blobs.
- **Blob Detection and Counting:** After the watershed, blob detection (`bwlabel`) and area calculations are performed on the result to filter and count blobs based on size.
- **Visualization and Saving:** The script then overlays the filtered blob mask in red on the original image and saves the result.

This modified script includes watershed segmentation to better separate touching or overlapping objects before counting and highlighting them. Remember to adjust the parameters based on the specifics of your images and desired outcomes.

Error in watershed(-threshImage, mask = threshImage > 0) :
  unused argument (mask = threshImage > 0) 40

The error you're encountering suggests there was a misunderstanding on my part regarding the parameters supported by the `watershed` function in `EBImage`. The `watershed` function in `EBImage` does not have a `mask` argument, which is common in other implementations of the watershed algorithm. Let's correct the approach to perform watershed segmentation without using a mask parameter directly in the function call.

Corrected Approach for Watershed Segmentation

You can apply the watershed algorithm directly to the negative of the thresholded image. If you need to apply it within a specific mask, consider modifying the image to set values outside the mask to a high value (or low, depending on how you want the watershed to behave) before applying the watershed. However, simply inverting the thresholded image and applying the watershed might suffice for many cases.

Here's the revised part of the script for applying watershed segmentation:

R   Code kopiëren

```r
# Invert the thresholded image for watershed analysis
invThreshImage <- -threshImage
# Apply the watershed algorithm
wsResult <- watershed(invThreshImage)

# Since we're not using a binary mask to limit the watershed,
# you might need to post-process wsResult to remove any undesired segmentation

# Continue with blob analysis and visualization as before
# ...

# Save the watershed result
writeImage(wsResult, 'watershedResult.jpg')
```

Additional Notes:

- **Watershed Application:** The `watershed` function is used on the inverted threshold image. The inversion (`-threshImage`) is a common approach when you want the watershed to treat dark regions as valleys to be flooded.
- **Post-processing:** Depending on your results, you might find it useful to apply additional processing after the watershed to refine the segmentation. This could involve removing small segments or applying a mask manually if there are areas you want to exclude.

Full Script Adjustment:

Replace the watershed segment of your script with the corrected approach above. The rest of your script, including the thresholding, saving images, blob detection, and visualization, remains applicable. This correction should eliminate the error and allow you to proceed with the watershed analysis as part of your image processing workflow.

Error in watershed(invThreshImage) :
  argument "priority" is missing, with no default 41

> wsResult <- watershed(priorityMap, threshImage)
Error: Expecting a four-dimensional array

The error messages you're encountering suggest there's some confusion about how the `watershed` function is used within the `EBImage` package in R. Let's clarify the usage of `watershed` and provide a correct approach to apply it for segmenting your image.

The `watershed` function in `EBImage` is designed to segment the image into regions based on the intensity gradient. The function requires a "priority map" where the segmentation process starts from the highest priority (typically the brightest pixels) and floods towards lower priority areas. The common practice for simple cases is to use the image itself or a transformed version of it as the priority map.

Correct Approach for Using Watershed in EBImage

When applying the watershed algorithm, ensure that your input is correctly formatted as a two-dimensional grayscale image or a three-dimensional color image. The error "Expecting a four-dimensional array" indicates that the function might be receiving input in an unexpected format. Let's correct this:

1. **Ensure the Image is Two-Dimensional:** For grayscale images, this is straightforward. For color images, you might need to convert them to grayscale or select a single channel.

2. **Use the Correct Input for Watershed:** The priority map should guide the segmentation. For simple cases, using the negative of the thresholded image as the priority map can work, but make sure the input is correctly formatted.

Here's a revised snippet:

R Code kopiëren

```R
library(EBImage)

# Assuming threshImage is your binary image from thresholding
# Convert it to a format suitable for watershed if it's not already
priorityMap <- -threshImage  # Using the negative image as priority

# Ensure priorityMap is a two-dimensional grayscale image
if (length(dim(priorityMap)) != 2) {
  priorityMap <- channel(priorityMap, "gray")
}

# Apply watershed
wsResult <- watershed(priorityMap)

# Continue with your analysis...
```

Tips for Successful Watershed Segmentation

- **Preprocessing:** The quality of watershed segmentation heavily depends on the preprocessing steps. Ensure your priority map highlights the regions of interest effectively.
- **Priority Map:** Experiment with different transformations of your image to use as a priority map. Sometimes, a combination of smoothing and gradient computation can provide better segmentation results.

If Problems Persist

If you continue facing issues, it might be helpful to revisit the documentation or examples for the `watershed` function in `EBImage` to ensure that there haven't been any changes or updates to the function's requirements. Additionally, consider validating the dimensions and type of your input images just before applying the watershed to catch any unexpected transformations.

Error in watershed(priorityMap) :
argument "priority" is missing, with no default 42

Given the error message you're encountering, it seems there's a need to clarify the correct usage of the `watershed` function within `EBImage`. The `watershed` function indeed requires a priority argument, which determines the order in which pixels are flooded. This priority map is crucial for guiding the watershed segmentation process.

Let's address this by ensuring we provide both the image and a priority map correctly to the `watershed` function. The priority map often comes from the gradient or the inverse of the image you want to segment because you want the watershed algorithm to start from the highest priority (brightest pixels in the priority map) regions.

Corrected Use of Watershed

First, if you haven't already, ensure your `EBImage` package is up to date, as the interface can change. Here's how you should structure your call to `watershed`:

R Code kopiëren

```r
library(EBImage)

# Assuming GImage is your pre-processed image
# First, let's create a priority map. A common approach is to use the gradient or the nega

# If GImage is not already a grayscale image, convert it to grayscale
if (length(dim(GImage)) == 3) {
  GImage <- channel(GImage, "gray")
}

# Create a priority map. Here, we use the Sobel operator to find edges, which are high-pri
```

```
priorityMap <- sobel(GImage)

# The watershed algorithm is applied to the binary image, guided by the priority map.
# The binary image guides where segmentation can occur, while the priority map defines the
wsResult <- watershed(threshImage, priorityMap)

# Now, wsResult contains the watershed segmentation result.
# Optionally, you can visualize or save this result.
writeImage(wsResult, "watershedResult.jpg")
```

This approach uses the Sobel operator to generate a priority map based on the edges detected in the grayscale version of the pre-processed image. The edges are considered high priority for the watershed segmentation, meaning the algorithm will start flooding from these regions.

Notes

- **Priority Map:** It's essential for guiding the watershed algorithm. Adjustments to the priority map can significantly affect the segmentation outcome.
- **Visualization:** After applying the watershed, it's a good practice to visualize the result to ensure it meets your expectations before proceeding with further analysis or saving the result.
- **Debugging:** If the segmentation does not work as expected, consider adjusting the preprocessing steps or the method used to generate the priority map. The goal is to have clear demarcations of regions in the priority map for effective segmentation.

This corrected approach should align with the functionality provided by the `EBImage` package for performing watershed segmentation and address the issue related to the missing "priority" argument.

Watershed did not work, trying something else + refeeding code to stay on track

43

```
# Read the image
image <-
readImage('E:/V2/
1.png') # Replace with your image path

# Convert to grayscale if necessary
#image <- channel(image, "gray")

# Enhance contrast (optional)
Eimage <- equalize(image)
FImage <- normalize(image)  # Linear contrast stretching
GImage <- gblur(FImage, sigma=1.1)  # Gaussian blur for noise
reduction

# Threshold the image
threshImage <- thresh(GImage, w=7, h=7, offset=0.035)
```

```
# Save the thresholded image
writeImage(threshImage, "thresholdedImage.jpg")
writeImage(Eimage, "EImage.jpg")
writeImage(FImage, "FImage.jpg")
writeImage(GImage, "GImage.jpg")

# Detect blobs (connected components)
blobs <- bwlabel(threshImage)

# Calculate area for each blob
blobAreas <- table(blobs)

# Filter blobs with size between 20 and 50 pixels
eligibleBlobs <- names(blobAreas)[blobAreas > 10 & blobAreas <=
80]
eligibleBlobMask <- blobs %in% eligibleBlobs

# Count the blobs
numBlobs <- max(blobs)

count <- sum(blobAreas > 10 & blobAreas < 80)

# Print the result
print(paste("Number of elements above 10 and below 80:", count))

# Print the number of blobs
print(paste("Number of blobs detected:", numBlobs))

# Assuming 'blobs' is already computed


# Convert the original image to RGB if it's grayscale
if (length(dim(image)) == 2) {
  colorImage <- abind(image, image, image, along = 3)
} else {
  colorImage <- image
}

# Create a mask for filtered blobs
filteredBlobsRedMask <- array(0, dim = dim(colorImage))
filteredBlobsRedMask[,,1][eligibleBlobMask] <- 1 # Red channel

# Overlay the red mask on the original image
colorImage[filteredBlobsRedMask > 0] <- 1

# Save the image with filtered blobs highlighted in red
writeImage(colorImage, 'imageWithFilteredBlobs.jpg')
```
after thresholding some blobs are confluent. How to divorce them?

To separate confluent blobs after thresholding, especially when they are touching or overlapping, you can use morphological operations and the watershed segmentation technique. The watershed technique is particularly effective for this purpose as it can help to delineate the boundaries between adjacent blobs by treating the grayscale image as a topographic surface and "flooding" it from specific seed points.

Given your workflow, you already have a thresholded image (`threshImage`). To apply the watershed technique effectively, you may need to:

1. **Identify Seed Points:** Determine the centers of the blobs that you want to separate. This can often be done by computing the distance transform of the binary image and then finding local maxima, which serve as seeds for the watershed.

2. **Prepare the Image for Watershed:** Use the gradient or the distance transform of the binary image as input for the watershed, as it requires a grayscale image where the blobs to be separated are represented by valleys.

Step-by-Step Guide to Separating Blobs

Here's how you can integrate these steps into your R script:

R Code kopiëren

```r
library(EBImage)

# Assuming GImage has been preprocessed and threshImage obtained
# Compute the distance transform of the binary thresholded image
distTransform <- distanceMap(threshImage)

# Find local maxima as seed points for the watershed
seeds <- localMaxima(distTransform, threshold = 0.1)

# Convert seeds to a binary image the same size as threshImage
seedImage <- seeds > 0

# Perform watershed segmentation
wsResult <- watershed(distTransform, seedImage)

# Optionally, visualize the watershed result
display(wsResult)

# Continue with blob analysis using wsResult...
```

Notes:

- Distance Transform (`distanceMap`): This function computes the distance of each foreground pixel to the nearest background pixel, which helps in identifying the centers of blobs.
- Local Maxima (`localMaxima`): Identifies seed points for the watershed. Adjust the `threshold` parameter based on the scale of your features.
- Watershed Segmentation (`watershed`): Applies the watershed algorithm using the distance transform as the surface to "flood" and the seeds as starting points. Note: You might need to adjust how seeds are generated and used based on your specific version of `EBImage` and its functionality.

Adjustments and Considerations:

- Fine-tuning Parameters: The effectiveness of this method depends on the correct identification of seed points and the characteristics of the distance transform. You might need to experiment with thresholds and other parameters.
- Post-Processing: After applying watershed, you may need to apply additional processing steps to refine the segmentation, such as filtering small artifacts or merging very small segments with adjacent larger ones.

This approach should help in separating confluent blobs in your images, allowing for more accurate analysis and counting of individual features.

Error in distanceMap(threshImage) : could not find function "distanceMap" 44

The error message indicates that the `distanceMap` function is not recognized, which may be due to a misunderstanding of the function's availability in `EBImage` or another package. In `EBImage`, the equivalent function for computing the distance transform of a binary image is `distmap`, not `distanceMap`. My apologies for the confusion. Let's correct this and proceed with the watershed segmentation using `distmap`.

Here's how you can adjust the script to incorporate the correct function for the distance transform and proceed with the watershed segmentation:

Corrected Script for Separating Confluent Blobs

R Code kopiëren

```
library(EBImage)

# Assuming threshImage is your binary thresholded image
# Compute the distance transform of the binary thresholded image
distTransform <- distmap(threshImage)

# Convert the distance transform to a binary image by finding local maxima
# This step requires identifying seed points, which can be challenging directly in EBImage
# As an alternative, let's use the thresholded image directly for simplicity

# Invert the distance transform for watershed; high values become low and vice versa
```

```
invDistTransform <- max(distTransform) - distTransform

# Apply watershed segmentation on the inverted distance transform
# EBImage's watershed function doesn't require seed points as input but operates on the en
wsResult <- watershed(invDistTransform)

# Visualize the watershed result
display(wsResult)

# Continue with your analysis...
```

Additional Notes:

- Distance Transform: `distmap` computes the distance of each foreground (white) pixel to the nearest background (black) pixel.
- Watershed Segmentation: `watershed` is applied to the inverted distance transform. In `EBImage`, the `watershed` function automatically handles the segmentation based on the grayscale values, treating darker values as valleys where water starts filling.
- Visualization and Analysis: After applying `watershed`, you have a segmented image where confluent blobs are separated. You can then proceed with further analysis, such as counting or measuring properties of individual blobs.

Please ensure that you adjust the parameters and steps according to your specific needs and the characteristics of your images. This approach should help in separating touching or overlapping objects in your binary images.

Error in UseMethod("distmap") :
 no applicable method for 'distmap' applied to an object of class "c('Image', 'array', 'structure', 'vector')" 45

The error you're encountering with `distmap` suggests a compatibility issue with the input image type in `EBImage`. It seems there's been a confusion regarding the availability or naming of the function within `EBImage` for calculating the distance map. If `distmap` is not recognized, it indicates a misunderstanding or an update in the package that I wasn't aware of.

Given this situation, the goal is still to perform watershed segmentation to separate confluent blobs, but without a direct `distanceMap` or `distmap` function from `EBImage`, we'll need to adjust our approach. Unfortunately, `EBImage` does not directly provide a distance transform function under those names, and my previous instructions may have led to confusion.

Alternative Approach for Watershed Without `distmap`

Since directly using a distance map for watershed segmentation seems problematic due to the function availability, let's focus on an alternative approach using the tools available in `EBImage`. We'll proceed with the watershed segmentation based on the gradient or the original image itself.

r Code kopiëren

```r
library(EBImage)

# Assuming GImage has been pre-processed
# Prepare the image for watershed segmentation
# Inverting the image so that the peaks become valleys
invImage <- max(GImage) - GImage

# Apply watershed segmentation
wsResult <- watershed(invImage)

# Visualize the result
display(wsResult)
```

Key Points

- **Watershed Segmentation:** This example directly uses the inverted pre-processed image for watershed segmentation. The idea is that areas between blobs will have higher values, acting as barriers.
- **Visualization:** The `display` function from `EBImage` is used to visualize the segmentation result.

Note

This simplified approach doesn't use a distance map for seed detection and might not perfectly separate very close or touching objects as effectively as the distance map-based method would. However, it still applies the watershed algorithm to segment the image based on intensity gradients.

For complex segmentation tasks where precise control over the segmentation seeds is necessary, considering additional image processing libraries or external tools that support comprehensive watershed segmentation with custom seeds might be beneficial. Python's scikit-image or OpenCV libraries offer extensive support for such tasks and might serve as valuable resources for complex image processing workflows.

```
> wsResult <- watershed(invImage)
Error in watershed(invImage) :
  argument "priority" is missing, with no default
```

46

Given the persistent issues with the `watershed` function usage in `EBImage` and my previous incorrect references, let's clarify the correct usage of watershed segmentation in R, focusing on the tools available. My previous suggestions overlooked the exact requirements for the `watershed` function in the context of `EBImage`. If `EBImage`'s `watershed` function is not behaving as expected or if there's confusion about its parameters, it suggests a need for a direct and accurate approach or exploring alternative methods.

Unfortunately, without the correct support for a `distmap` function in `EBImage` and the correct usage of `watershed`, achieving the desired segmentation to separate confluent blobs becomes challenging within the constraints of this specific library's version and functionality as described.

Alternative Approach for Complex Image Segmentation

Given the limitations encountered with `EBImage` for this task, consider using alternative tools or libraries that might offer more straightforward or well-documented support for advanced segmentation tasks like watershed with custom priority maps. For R users, transitioning to a Python-based workflow for this part of the image processing might be worth considering, given the robust support in libraries like `scikit-image` or `OpenCV` for Python.

Python Example with scikit-image

If transitioning to Python for this task is an option, here's a quick example of how you might use `scikit-image` to perform watershed segmentation with more control:

python Code kopiëren

```python
from skimage import io, filters, morphology, segmentation
import numpy as np

# Load the image
image = io.imread('path/to/image.png')

# Assuming image preprocessing is already done, and you have a binary image
binary_image = image > filters.threshold_otsu(image)  # Example thresholding

# Compute the distance transform
distance = morphology.distance_transform_edt(binary_image)

# Generate markers for watershed
local_maxi = morphology.local_maxima(distance)
markers = morphology.label(local_maxi)

# Perform watershed segmentation
ws_labels = segmentation.watershed(-distance, markers, mask=binary_image)
```

```
# Save or display the result
io.imsave('watershed_result.png', ws_labels)
```

Recommendations

- **Python for Advanced Image Processing:** For complex image segmentation tasks, Python's ecosystem provides powerful tools with extensive documentation and community support.
- **Seek Updated Documentation:** If sticking with R and `EBImage` is necessary, I recommend seeking the most up-to-date documentation for `EBImage` or consulting community forums for advice on advanced segmentation tasks. The functionality and available functions in `EBImage` (or any library) can change, and direct documentation or examples from the developers will be the most reliable source of information.

I apologize for any confusion my previous responses may have caused and hope this guidance offers a clearer path forward for your image segmentation needs.

Distance mapping did not work, trying something else

can I count blobs with a give size as two blobs, or 3 ? in R (47)

Yes, in R, you can count blobs of a given size as two or three blobs (or any other number), based on your specific criteria. This involves analyzing the size (e.g., area) of each detected blob and then deciding how to count it based on its size relative to a predefined threshold. You might want to do this if you know that blobs of a certain size typically represent multiple, closely spaced objects that the segmentation process failed to separate.

To implement this, you'll first need to detect and measure the blobs, as you've done previously. Then, you can apply your custom logic to count larger blobs as multiple instances based on their size.

Here's an example approach:

Step 1: Detect and Measure Blobs

You've already got this part in your script where you use `bwlabel` to label blobs and then calculate their areas.

r   Code kopiëren

```r
blobs <- bwlabel(threshImage)  # Label blobs
blobAreas <- table(blobs)  # Calculate area of each blob
```

Step 2: Apply Custom Counting Logic

Define thresholds and logic to count larger blobs as multiple instances.

r Code kopiëren

```r
# Initialize total blob count
totalBlobs = 0

# Loop through each blob area
for (area in blobAreas) {
  if (area > lowerThreshold && area <= upperThreshold) {
    # If the blob is within a certain size range, count it as 2 (or any other logic)
    totalBlobs <- totalBlobs + 2
  } else if (area > upperThreshold) {
    # For even larger blobs, you might count them as 3 or more
    totalBlobs <- totalBlobs + 3
  } else {
    # Count normally
    totalBlobs <- totalBlobs + 1
  }
}

# Print the adjusted blob count
print(paste("Adjusted number of blobs:", totalBlobs))
```

Customizing Your Logic

- Define `lowerThreshold` and `upperThreshold` based on the size range of blobs you want to count differently. These thresholds should be set according to your domain knowledge or empirical observations of your image data.
- **Adjust the counting logic** within the loop based on how you want to count blobs of different sizes. The example above is simplistic and assumes blobs within a certain size range should count as double, and blobs above a larger threshold should count as triple. You can refine this logic based on more complex criteria or additional size thresholds.

Important Note

This approach requires careful calibration of the size thresholds (`lowerThreshold` and `upperThreshold`) and the counting logic to accurately reflect the characteristics of the objects you're analyzing in your images. It's a somewhat heuristic method, so validating the approach against known standards or manual counts is advisable to ensure accuracy.

i want a list of all blobs, area, and counted value: as check 48

To create a list (or more accurately, a data frame in R) that includes all blobs, their area, and the counted value (how many times each blob is counted based on its area), you can follow this approach. This not only allows you to count blobs as multiple instances based on their size but also provides a clear overview for verification.

Here's how you can modify and expand upon your existing script to accomplish this:

Expanded Script with Data Frame Output

r Code kopiëren

```r
library(EBImage)

# Assuming 'blobs' and 'blobAreas' are already defined in your workspace
# blobs <- bwlabel(threshImage)
# blobAreas <- table(blobs)

# Initialize an empty data frame to store blob details
blobDetails <- data.frame(blobID = integer(), area = integer(), countedValue = integer())

# Define your thresholds
lowerThreshold <- 20  # Define according to your criteria
upperThreshold <- 50  # Define according to your criteria

# Populate the data frame with blob details and counted values
for (i in seq_along(blobAreas)) {
  area <- blobAreas[i]
  countedValue <- ifelse(area > lowerThreshold & area <= upperThreshold, 2,
                         ifelse(area > upperThreshold, 3, 1))

  # Append to the data frame
  blobDetails <- rbind(blobDetails, data.frame(blobID = names(blobAreas)[i], area = area,
}

# Print the blob details data frame
print(blobDetails)

# Calculate the total count based on the counted values
totalBlobs <- sum(blobDetails$countedValue)

# Print the adjusted total blob count
print(paste("Adjusted number of blobs:", totalBlobs))
```

Explanation

- Data Frame Creation: A data frame `blobDetails` is initialized to store the ID, area, and counted value for each blob.
- Loop Through Blobs: The script loops through each blob, calculating its area and determining its counted value based on the predefined thresholds (`lowerThreshold` and `upperThreshold`).
- Populate Data Frame: For each blob, a new row is added to the `blobDetails` data frame with the blob's ID, area, and how many times it should be counted (`countedValue`).
- Calculate Total Count: The total count of blobs, adjusted based on their sizes, is calculated by summing up the `countedValue` column of the data frame.

- Verification and Checks: The `blobDetails` data frame serves as a detailed record for verification, allowing you to check the area and counted value of each blob.

This approach provides a structured and transparent way to adjust the count of blobs based on their size, along with a comprehensive list for verification and checks. Adjust the `lowerThreshold` and `upperThreshold` values according to your specific requirements to accurately reflect the characteristics of the objects in your images.

i want the size of the largest and smallest blob 49

To find the size of the largest and smallest blob detected in your image, you can utilize the `blobAreas` calculation from the `table(blobs)` output, which you've already computed in your workflow. The `blobAreas` variable contains the area (in terms of the number of pixels) of each labeled blob.

Here's how you can determine the sizes of the largest and smallest blobs:

Finding the Largest and Smallest Blob Sizes

First, ensure you've identified and labeled blobs in your image and calculated their areas as shown in previous steps:

r Code kopiëren

```r
# Assuming 'blobs' has been computed with bwlabel
# blobAreas <- table(blobs)
```

Now, to find the sizes of the largest and smallest blobs:

r Code kopiëren

```r
# Convert blobAreas to a numeric vector (excluding the background which is usually labeled
blobSizes <- as.numeric(blobAreas[names(blobAreas) != 0])

# Find the size of the largest blob
largestBlobSize <- max(blobSizes)

# Find the size of the smallest blob
smallestBlobSize <- min(blobSizes)

# Print the results
print(paste("Size of the largest blob:", largestBlobSize))
print(paste("Size of the smallest blob:", smallestBlobSize))
```

Explanation:

- **Blob Sizes:** The `blobSizes` vector contains the area for each blob detected in the image, excluding the background (which may be labeled as `0` and should not be considered a blob).
- **Largest and Smallest Blob:** The `max` and `min` functions are used to find the largest and smallest values in the `blobSizes` vector, respectively.

This approach will give you the sizes of the largest and smallest blobs in terms of the number of pixels they contain. This information can be very useful for understanding the range of object sizes in your images and can also help in setting thresholds for filtering blobs based on their size.

if a blob is part of a bigger blob, is it counted twice? 50

In the context of blob detection and analysis, whether a smaller blob "part of" a bigger blob is counted twice depends on how you define and detect blobs in your image processing workflow. Generally, the approach to counting depends on the segmentation and labeling steps. Here are common scenarios:

Scenario 1: Distinct Blob Detection

- **Independent Blobs:** If each blob is detected and labeled independently (e.g., using a binary threshold and connected component labeling), then each blob is counted once based on its label. In this case, smaller blobs within a larger blob boundary that are not connected are counted separately.
- **Overlap Not Considered:** If the smaller blob is entirely within the larger blob but is disconnected (e.g., a small object on a larger distinct background object), both are counted separately due to different labels.

Scenario 2: Nested or Overlapping Blobs

- **Nested Detection:** If your detection algorithm or processing steps do not differentiate between nested or overlapping structures, a smaller blob within a larger one might not be identified separately. The count would then reflect only the larger encompassing blob.
- **Special Handling for Overlap:** If you use techniques that can identify and separate overlapping or nested blobs (such as watershed segmentation), then each distinct region is labeled and counted separately, even if one is within another.

Handling Nested or Part-of Relationships

To explicitly handle scenarios where smaller blobs are part of larger ones, and you wish to count them differently, you might:

- **Adjust Detection/Segmentation:** Use advanced segmentation techniques like watershed to separate closely touching or overlapping blobs.
- **Post-Processing Logic:** Implement custom logic in your analysis to adjust counts based on size, proximity, or hierarchical relationships identified between blobs.

Example: Adjusting Counts Based on Hierarchy

If you have a way to identify when one blob is part of another (e.g., through spatial analysis or hierarchy detection post-segmentation), you can adjust your counting accordingly. This might involve:

- **Spatial Analysis:** Determining if the centroid of a smaller blob lies within the boundary of a larger blob.
- **Count Adjustment:** Programmatically deciding that in such cases, the smaller blob's presence adjusts the count of the larger blob, or perhaps not counting the smaller blob separately.

Summary

- **Default Counting:** Without specific handling, blobs are counted based on their detection and labeling, where each label corresponds to a separate count.
- **Custom Handling Needed:** To adjust counts for nested blobs or parts of larger blobs, you'll need to implement additional logic based on your analysis goals and the specific characteristics of your image data.

In practical terms, without additional context or specific handling, blobs are counted based on how they are detected and labeled, not inherently twice due to being part of a larger blob.

```
# Read the image
image <-
readImage('E:/V2/1
1.png') # Replace with your image path

# Convert to grayscale if necessary
#image <- channel(image, "gray")

# Enhance contrast (optional)
Eimage <- equalize(image)
FImage <- normalize(image)  # Linear contrast stretching
GImage <- gblur(FImage, sigma=1.1)  # Gaussian blur for noise
reduction

# Threshold the image
threshImage <- thresh(GImage, w=7, h=7, offset=0.025)

# Save the thresholded image
writeImage(threshImage, "thresholdedImage.jpg")
writeImage(Eimage, "EImage.jpg")
writeImage(FImage, "FImage.jpg")
writeImage(GImage, "GImage.jpg")
```

(51)

```
# Detect blobs (connected components)
blobs <- bwlabel(threshImage)

# Calculate area for each blob
blobAreas <- table(blobs)

# Filter blobs with size between 20 and 50 pixels
eligibleBlobs <- names(blobAreas)[blobAreas > 10 & blobAreas
<=340]
eligibleBlobMask <- blobs %in% eligibleBlobs

# Count the blobs
numBlobs <- max(blobs)


# Loop through each blob area
for (area in blobAreas) {
  if (area > 35 && area <= 70) {
    # If the blob is within a certain size range, count it as 2 (or any other
logic)
    totalBlobs <- totalBlobs + 2
  } else if (area > 70 && area <= 105) {
    # For even larger blobs, you might count them as 3 or more
    totalBlobs <- totalBlobs + 3
  } else if (area > 105 && area <= 140) {
    # For even larger blobs, you might count them as 3 or more
    totalBlobs <- totalBlobs + 4
  } else if (area > 140 && area <= 175) {
    # For even larger blobs, you might count them as 3 or more
    totalBlobs <- totalBlobs + 5
  } else if (area > 175 && area <= 210) {
    # For even larger blobs, you might count them as 3 or more
    totalBlobs <- totalBlobs + 6
  } else if (area > 245 && area <= 280) {
    # For even larger blobs, you might count them as 3 or more
    totalBlobs <- totalBlobs + 7
  } else if (area > 280 && area <= 315) {
    # For even larger blobs, you might count them as 3 or more
    totalBlobs <- totalBlobs + 8
  } else if (area > 315) {
     # For even larger blobs, you might count them as 3 or more
     totalBlobs <- totalBlobs + 9
  } else {
    # Count normally
    totalBlobs <- totalBlobs + 1
  }
}

# Print the result
print(paste("Number of elements above 10 and below 80:",
```

Manually extrapolating blob size correction to save time + feeding the script to work further from there on

# Print the number of blobs
print(paste("Number of blobs detected:", numBlobs))

Assuming 'blobs' is already computed

# Convert the original image to RGB if it's grayscale
if (length(dim(image)) == 2) {
  colorImage <- abind(image, image, image, along = 3)
} else {
  colorImage <- image
}

# Create a mask for filtered blobs
filteredBlobsRedMask <- array(0, dim = dim(colorImage))
filteredBlobsRedMask[,,1][eligibleBlobMask] <- 1 # Red channel

Overlay the red mask on the original image
colorImage[filteredBlobsRedMask > 0] <- 1

Save the image with filtered blobs highlighted in red
writeImage(colorImage, 'imageWithFilteredBlobs.jpg') exactly this script in its whole, looped through each image in a folder

To execute the entire provided script for each image in a folder, incorporating all steps from image reading and processing to saving the final images, you can wrap the code in a loop that iterates through all image files in the specified directory. Here's how you can do it:

R Script for Batch Processing Images in a Folder

r Code kopiëren

```r
library(EBImage)

# Define the input and output folders
inputFolder <- 'E:/V2/'
outputFolder <- 'E:/ProcessedImages/'

# Create the output folder if it doesn't exist
if (!dir.exists(outputFolder)) {
  dir.create(outputFolder)
}

# List all image files in the input folder (adjust pattern as needed for different file ty
imageFiles <- list.files(inputFolder, pattern = '\\.(png|jpg)$', full.names = TRUE)

# Process each image file
for (file in imageFiles) {
  # Read the image
```

```
image <- readImage(file)

# Image processing steps
Eimage <- equalize(image)
FImage <- normalize(image)  # Linear contrast stretching
GImage <- gblur(FImage, sigma = 1.1)  # Gaussian blur for noise reduction
threshImage <- thresh(GImage, w = 7, h = 7, offset = 0.025)

# Blob detection and counting
blobs <- bwlabel(threshImage)
blobAreas <- table(blobs)

eligibleBlobs <- names(blobAreas)[blobAreas > 10 & blobAreas <= 340]
eligibleBlobMask <- blobs %in% eligibleBlobs

totalBlobs <- 0
# Adjusted blob counting loop
for (area in blobAreas) {
  if (area > 35 && area <= 70) {
    totalBlobs <- totalBlobs + 2
  } else if (area > 70 && area <= 105) {
    totalBlobs <- totalBlobs + 3
  } else if (area > 105 && area <= 140) {
    totalBlobs <- totalBlobs + 4
  } else if (area > 140 && area <= 175) {
    totalBlobs <- totalBlobs + 5
  } else if (area > 175 && area <= 210) {
    totalBlobs <- totalBlobs + 6
  } else if (area > 245 && area <= 280) {
    totalBlobs <- totalBlobs + 7
  } else if (area > 280 && area <= 315) {
    totalBlobs <- totalBlobs + 8
  } else if (area > 315) {
    totalBlobs <- totalBlobs + 9
  } else {
    totalBlobs <- totalBlobs + 1
  }
}

# Assuming 'blobs' is already computed
# Convert the original image to RGB if it's grayscale
if (length(dim(image)) == 2) {
  colorImage <- abind(image, image, image, along = 3)
} else {
  colorImage <- image
}

# Create a mask for filtered blobs
filteredBlobsRedMask <- array(0, dim = dim(colorImage))
filteredBlobsRedMask[,,1][eligibleBlobMask] <- 1 # Red channel
```

```
    # Overlay the red mask on the original image
    colorImage[filteredBlobsRedMask > 0] <- 1

    # Construct output filenames
    baseName <- gsub(pattern = ".*\\/", replacement = "", x = file)
    baseName <- gsub(pattern = "\\.[a-z]+$", replacement = "", x = baseName)

    # Save the processed images
    writeImage(threshImage, paste0(outputFolder, baseName, '_thresholdedImage.jpg'))
    writeImage(EImage, paste0(outputFolder, baseName, '_EImage.jpg'))
    writeImage(FImage, paste0(outputFolder, baseName, '_FImage.jpg'))
    writeImage(GImage, paste0(outputFolder, baseName, '_GImage.jpg'))
    writeImage(colorImage, paste0(outputFolder, baseName, '_FilteredBlobs.jpg'))

    # Optionally, save the total blobs count to a text file
    cat(paste("Total blobs:", totalBlobs, "\n"), file = paste0(outputFolder, baseName, '_blo
  }

  cat("All images processed and saved.\n")
```

Notes:

- Folder Paths: Ensure that `inputFolder` and `outputFolder` reflect your actual directories.
- Image Types: The script currently targets `.png` and `.jpg` files. Adjust the `pattern` in `list.files()` for other formats as needed.
- Filename Handling: The script extracts the base filename from each path to construct output filenames. This approach removes the directory and file extension

Error in readPNG(x, ...) : libpng error: IDAT: incorrect data check 52

The error message you're encountering, `Error in readPNG(x, ...): libpng error: IDAT: incorrect data check`, typically indicates a problem with reading the PNG file due to possible corruption or an unsupported format feature by the `readPNG` function from the `png` package (implicitly used by `readImage` in `EBImage` when dealing with PNG files).

Here are several steps you can take to troubleshoot and potentially resolve this issue:

1. Verify File Integrity

- **Check the PNG File:** Ensure the PNG file is not corrupted. You can do this by trying to open it in an image viewer or editor. If the file doesn't open or shows errors in other software, it's likely corrupted.
- **Try Re-saving the Image:** If you can open the image in an editor (like GIMP, Photoshop, or even Paint), try re-saving it with a different name or converting it to a different format (e.g., JPEG) and

see if `readImage` can read the new file.

2. Update Your Packages

Ensure that your `EBImage` and its dependencies are up to date. Older versions might lack support for certain PNG features or have bugs that have been fixed in later versions.

```r
if (!requireNamespace("BiocManager", quietly = TRUE))
    install.packages("BiocManager")
BiocManager::install("EBImage")
```

3. Use Alternative Functions for Reading the Image

If updating doesn't help or the file is not corrupted, try using a different function to read the PNG file and then convert it to an `EBImage` image object:

- Using the `png` Package Directly:

```r
library(png)
library(EBImage)

# Read the PNG file
img <- readPNG('path/to/your/image.png')

# Convert to an EBImage Image object
image <- EBImage::Image(img)

# Now proceed with your processing...
```

- For JPEG or Other Formats, you might use the `jpeg` package or `imager` package similarly if `EBImage` directly fails.

4. Convert the File Format

If the PNG file is problematic and cannot be read directly by R, consider converting it to another format like JPEG. This can be done using image editing software or command-line tools like ImageMagick.

5. Manual Check and File Repair

- **File Repair:** For corrupted files, software tools designed to repair PNG files might resolve the issue. However, this approach can be hit or miss depending on the corruption extent.
- **Check for Consistency:** Ensure all images in the batch are consistent in terms of bit depth, color mode, and do not contain any special PNG features that might be unsupported.

By following these steps, you should be able to identify the cause of the issue and find a workaround to successfully process your images in R.

Error in readPNG(x, ...) : libpng error: IDAT: incorrect data check why? 53

The error `Error in readPNG(x, ...): libpng error: IDAT: incorrect data check` during the execution of `readPNG` or indirectly through `readImage` in R indicates a problem encountered by the underlying `libpng` library when reading the PNG file. This issue usually points to one of the following:

1. **Corrupted PNG File:** The most common reason for this error is that the PNG file itself is corrupted. Corruption can occur due to incomplete downloads, errors in saving from an image editing program, or issues during file transfer. The corruption might affect the IDAT chunk, which contains the actual image data.

2. **Unsupported PNG Features:** Occasionally, this error might arise if the PNG file uses certain features or compression methods that are not fully supported or expected by the version of `libpng` that your R environment is using.

Troubleshooting Steps:

1. **Verify the Image:** Try opening the PNG file in a different image viewer or editor to check if it's corrupted. If the file doesn't open or displays errors, it's likely corrupted.

2. **Re-save the Image:** If you can open the file in an image editor (like Photoshop, GIMP, or Paint.NET), try re-saving it with a different name or converting it to a different format (e.g., JPEG) and see if the issue persists when reading the new file in R.

3. **Update or Reinstall Packages:** Make sure your R packages (`png`, `EBImage`, etc.) and their dependencies are up to date. Sometimes, updating can resolve compatibility issues with file formats.

```r
# Update png package
install.packages("png")
# For EBImage, if you're using Bioconductor
if (!requireNamespace("BiocManager", quietly = TRUE))
    install.packages("BiocManager")
BiocManager::install("EBImage")
```

4. **Use Alternative Methods:** If the problem is specific to `readPNG` or `readImage`, try using alternative functions or packages to read the image. For instance, the `imager` package in R also supports reading various image formats, including PNG.

```r
# Example using the imager package
if (!requireNamespace("imager", quietly = TRUE))
    install.packages("imager")

library(imager)
image <- load.image('path/to/your/image.png')
```

5. **Convert the Image Outside R:** Use external tools like ImageMagick or online services to convert or repair the PNG file before reading it into R. This can help if the issue is due to unsupported features or minor corruption that such tools can handle.

6. **Check for File Integrity Issues:** If the file was downloaded from the internet, ensure it was downloaded completely and correctly. Incomplete downloads are a common source of file corruption.

By following these steps, you should be able to identify and mitigate the issue causing the `libpng error: IDAT: incorrect data check` error when processing PNG files in R.

Error in readPNG(x, ...) : libpng error: IDAT: incorrect data check R encoutered a fatal error 54

The error message `Error in readPNG(x, ...): libpng error: IDAT: incorrect data check` followed by R encountering a fatal error is indicative of a deeper issue with the PNG file you are trying to read. This problem arises from the `libpng` library, which is used by R (specifically the `png` package) to handle PNG files. The `IDAT` mentioned in the error refers to the image data chunk within the PNG file format, and "incorrect data check" suggests a corruption or an unexpected format that `libpng` cannot correctly interpret or recover from.

Given that this error is causing R to crash, it's important to approach the solution carefully:

1. **Verify File Integrity**:

- Try opening the PNG file with different image viewers or editors to confirm it's not corrupted.
- If the file cannot be opened or displays errors in other software, it's likely corrupted.

2. **Re-save or Convert the File**:

- If you can open the file in an image editing program, re-save it under a new name or convert it to a different format (e.g., JPEG), then try reading it again in R.

3. **Update R and Related Packages**:

- Ensure your R environment and all related packages (`png`, `EBImage`) are up to date. An outdated `libpng` version might lack support for certain PNG features.

r Code kopiëren

```r
update.packages()
if (!requireNamespace("BiocManager", quietly = TRUE))
    install.packages("BiocManager")
BiocManager::install("EBImage")
```

4. **Use Alternative Libraries**:

- If `readPNG` consistently fails, consider using alternative R packages that can read PNG files. For example, the `imager` package offers comprehensive image processing capabilities and might handle the file differently.

r Code kopiëren

```r
if (!require("imager")) install.packages("imager")
library(imager)
img <- load.image("path/to/your/image.png")
```

5. **External Validation and Conversion**:

- Use external tools like ImageMagick to validate or convert the PNG file. ImageMagick can provide detailed information about potential issues with the file or successfully convert it to a different format that R can handle without issues.

bash Code kopiëren

```bash
magick convert corrupt.png fixed.png
```

6. **Isolate and Test**:

- If possible, test reading a different PNG file in R to ensure the issue is isolated to specific files and not a broader problem with your R setup.

7. **System Check**:

- Since R encountered a fatal error, it's also wise to check your system's health. Ensure there's enough memory available and that your system's disk integrity is good, especially if this issue occurs with multiple files or suddenly started happening.

If the Problem Persists

If after these steps R continues to crash when attempting to read PNG files, consider seeking help from the R community (e.g., R-help mailing list, Stack Overflow) with detailed information about your session (`` `sessionInfo()` ``), the specific file(s) causing the issue, and the steps you've already tried. Sharing this information can help diagnose whether it's a problem with the file, R's configuration, or a bug in the underlying libraries.

Over here a used an earlier generated snippet to finalise the code shown in supplemental 1
