# Supplementary material for: Adaptive Optics Image Analysis Using Generative AI (GPT-4) Scripting: Exploratory Study
Source: JMIR Form Res. 2026 Sep 14;10:e94906. doi: 10.2196/94906 (PMC13620430; doi:10.2196/94906)
Supplement: Supplementary file 2 [file formative_v10i1e94906_app1.docx]

library(EBImage)

library(imager)

library(abind)

library(spatstat)

\# Define the input and output folders

\# Specify the folder containing the images

folderPath <- 'E:/V2' # Replace with your folder path

\# Get a list of image file paths

image_files <- list.files(folderPath, pattern = '\\.(jpg|jpeg|png)$', full.names = TRUE)

\# Initialize a data frame to store results

results <- data.frame(fileName = character(), blobCount = integer(), stringsAsFactors = FALSE)

\# Process each image file

for (file in image_files) {

image <- readImage(file)

\# Apply your image processing steps here

\# ... (use your existing image processing code)

\# Convert to grayscale if necessary

#image <- channel(image, "gray")

\# Enhance contrast (optional)

Eimage <- equalize(image)

FImage <- normalize(image) # Linear contrast stretching

GImage <- gblur(FImage, sigma=1.1) # Gaussian blur for noise reduction

\# Threshold the image

threshImage <- thresh(GImage, w=4.5, h=4.5, offset=0.043)

\# Detect blobs (connected components)

blobs <- bwlabel(threshImage)

\# Calculate area for each blob

blobAreas <- table(blobs)

\# Calculate area for each blob

blobAreas <- table(blobs)

\# Filter blobs with size between 20 and 50 pixels

eligibleBlobs <- names(blobAreas)[blobAreas > 10 & blobAreas <=240]

eligibleBlobMask <- blobs %in% eligibleBlobs

\# Count the blobs

totalBlobs = 0

\# Loop through each blob area

for (area in blobAreas) {

if (area > 35 && area <= 70) {

\# If the blob is within a certain size range, count it as 2 (or any other logic)

totalBlobs <- totalBlobs + 2

} else if (area > 10 && area <= 35) {

\# For even larger blobs, you might count them as 3 or more

totalBlobs <- totalBlobs + 1

} else if (area > 70 && area <= 105) {

\# For even larger blobs, you might count them as 3 or more

totalBlobs <- totalBlobs + 3

} else if (area > 105 && area <= 140) {

\# For even larger blobs, you might count them as 3 or more

totalBlobs <- totalBlobs + 4

} else if (area > 140 && area <= 175) {

\# For even larger blobs, you might count them as 3 or more

totalBlobs <- totalBlobs + 5

} else if (area > 175 && area <= 210) {

\# For even larger blobs, you might count them as 3 or more

#totalBlobs <- totalBlobs + 6

#} else if (area > 245 && area <= 280) {

\# For even larger blobs, you might count them as 3 or more

#totalBlobs <- totalBlobs + 7

\# } else if (area > 280 && area <= 315) {

\# For even larger blobs, you might count them as 3 or more

\# totalBlobs <- totalBlobs + 8

\# } else if (area > 315) {

\# For even larger blobs, you might count them as 3 or more

#totalBlobs <- totalBlobs + 9

} else {

\# Count normally

totalBlobs <- totalBlobs + 0

}

}

\# Print the number of blobs

print(paste("Number of blobs detected:", totalBlobs))

\# Assuming 'blobs' is already computed

\# Convert the original image to RGB if it's grayscale

if (length(dim(image)) == 2) {

colorImage <- abind(image, image, image, along = 3)

} else {

colorImage <- image

}

\# Create a mask for filtered blobs

filteredBlobsRedMask <- array(0, dim = dim(colorImage))

filteredBlobsRedMask[,,1][eligibleBlobMask] <- 1 # Red channel

\# Overlay the red mask on the original image

colorImage[filteredBlobsRedMask > 0] <- 1

\# Save the image with filtered blobs highlighted in red

writeImage(colorImage, 'imageWithFilteredBlobs.jpg')

\# Count the blobs

\# Annotate the image with the number of blobs

\# Annotate the image with the number of blobs

\# Save the processed image

outputFileName <- paste0(folderPath, '/processed_', basename(file))

writeImage(colorImage, outputFileName)

\# Store the result in the data frame

results <- rbind(results, data.frame(fileName = basename(file), blobCount = totalBlobs))

}

\# Print the results

print(results)
