# Supplementary material for: Adaptive Optics Image Analysis Using Generative AI (GPT-4) Scripting: Exploratory Study
Source: JMIR Form Res. 2026 Sep 14;10:e94906. doi: 10.2196/94906 (PMC13620430; doi:10.2196/94906)
Supplement: Supplementary file 3 [file formative_v10i1e94906_app4.docx]

a)

b)
